# The SibUS-In Finger Probe: An Alternative Device and Method for Ultrasound-Guided Injections

**DOI:** 10.1093/asjof/ojag046

**Published:** 2026-03-25

**Authors:** Audrey Melin, Diala Haykal, Jaoued Diboun, Hugues Cartier, Sebastien Garson, Jair Mauricio Cerón Bohórquez, Peter Velthuis, Leonie Schelke, Benjamin Ascher

## Abstract

Conventional ultrasound systems in aesthetic medicine are often bulky, single-hand operated, and poorly adapted to facial anatomy, limiting their practicality during injectable procedures. These ergonomic and workflow constraints have restricted the integration of ultrasound guidance into routine practice. The SibUS-In (safe injection by ultrasound) probe was specifically designed for aesthetic injections, integrating a high-frequency transducer onto the injector's fingertip to enable simultaneous palpation, scanning, and injection. This configuration restores 2-hand coordination and allows real-time visualization of facial structures while maintaining dexterity. In clinical use, the device combines ergonomic comfort, intuitive handling, and high-quality imaging that supports continuous vascular mapping and procedural control. The present article illustrates the practical application of the SibUS-In probe across different facial regions and procedural stages, highlighting its potential to facilitate ultrasound-guided injections. Further studies are warranted to evaluate quantitative outcomes and define standardized protocols for its broader clinical adoption.

**Level of Evidence**: 5 (Therapeutic) 
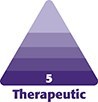

Ultrasound imaging is becoming an increasingly valuable tool in aesthetic medicine, offering real-time visualization capable of improving both precision and safety. Because aesthetic procedures continue to grow worldwide, they remain associated with potentially severe complications, such as vascular occlusion, skin necrosis, blindness, inflammatory reactions, or toxin-related asymmetries.^1,2^ Ensuring patient safety during injections has therefore become a central issue. High-resolution ultrasound and Doppler imaging have emerged as key tools to prevent injection-related complications through vascular mapping and real-time guidance, as well as to manage adverse events using targeted treatments such as hyaluronidase injection.^3-5^

However, most existing ultrasound systems were designed for radiology or emergency medicine. Their bulky linear form requires one hand to hold the probe, limiting dexterity and often demanding a second operator. These devices are poorly adapted to facial contours and interrupt the workflow, making them difficult to integrate into daily practice. High acquisition costs and the lack of dedicated training have further restricted their adoption in daily aesthetic practice, despite the growing awareness of ultrasound's safety benefits.

The SibUS-In (safe injection by ultrasound) probe (Think-In Tech, Paris, France) was specifically developed to overcome these limitations. It integrates a high-frequency curved transducer onto the index fingertip, enabling the injector to palpate, scan, and inject simultaneously. This design restores full autonomy to the practitioner and adapts naturally to facial anatomy. By combining tactile feedback with high-quality imaging, the device facilitates precise mapping, needle guidance, and controlled filler placement while maintaining ergonomic comfort. Although SibUS-In enhances real-time anatomical control, it is intended as a procedural adjunct rather than a diagnostic ultrasound device and does not replace radiological expertise. Its role is to support safer, more accurate injection techniques in aesthetic medicine—used here in its broadest sense to include both medical and surgical injectors—and it has the potential to become a standard in image-guided injection procedures.

## METHODS

This study did not require IRB approval because it was based on noninterventional clinical observation and documentation of standard aesthetic procedures performed in routine clinical practice. No investigational drug, device modification, or experimental protocol was applied. Written informed consent was obtained from all participating patients for the use of ultrasound imaging and the publication of anonymized photographs and ultrasound images. All procedures were conducted in accordance with the ethical principles of the Declaration of Helsinki.

### Technology

**Figure 1.**
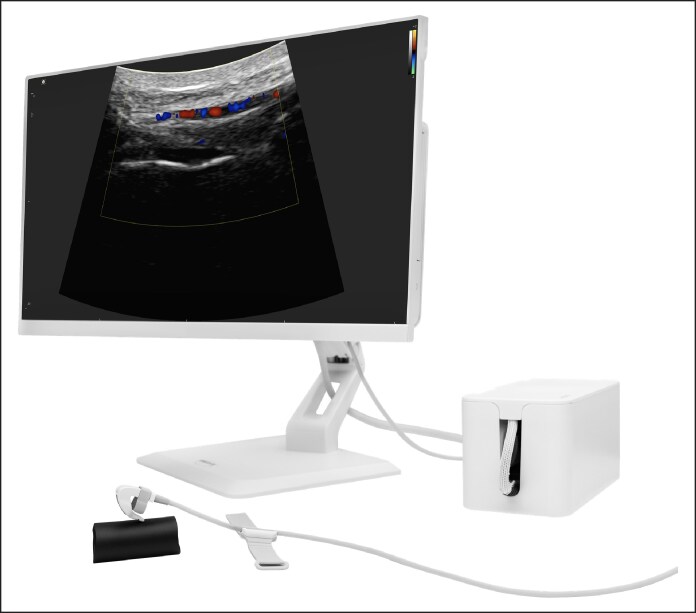
SibUS-In system comprising a 23-inch touchscreen monitor, processing unit, and digital ultrasound platform, with the wired fingertip-mounted probe visible in the foreground.

The SibUS-In probe consists of a miniaturized high-frequency (7-15 MHz) curved transducer mounted on an ergonomic fingertip support, most commonly on the index finger, as illustrated in Figure 1 and Supplemental Figure 1. Its compact and lightweight form allows close contact with the skin while preserving the operator's natural palpation and injection techniques. The remaining fingers act as a stabilizing tripod, maintaining control and preventing operator fatigue. The curved shape enables gentle tilting along complex facial regions such as the orbit, forehead, nose, deep pyriform space, and temples.

The probe provides both B-mode grayscale and Doppler imaging. It operates at frequencies between 7 and 15 MHz, ensuring high-resolution visualization of superficial and deeper structures. It also includes real-time image and video recording capabilities, enabling documentation of key procedural steps and vascular mapping when required. Optional preconfigured settings are available for special facial regions to optimize image quality, although a single setting can also be used throughout the procedure. Furthermore, its sectorial field of view offers wide-angle visualization, facilitating mapping of vascular and soft-tissue structures.

**Figure 2.**
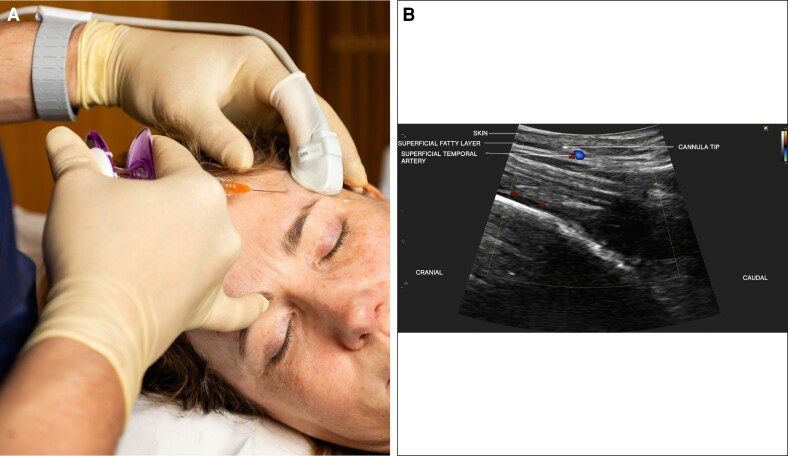
In a 57-year-old female patient, (A) the clinical view shows ultrasound-guided hyaluronic acid injection in the temple using the SibUS-In probe and a 25 G cannula introduced into the superficial fat. The probe is positioned on the index finger of the noninjecting hand, allowing simultaneous palpation and stable control during injection. (B) Doppler ultrasound imaging visualizes the 25 G cannula tip using an out-of-plane approach, in close relation to the superficial temporal artery, with both the superficial and deep temporal arteries clearly identified.

Typically worn on the nondominant index finger, it enables simultaneous palpation, scanning, and injection, overcoming one of the main limitations of conventional probes—as illustrated in Figure 2 and Supplemental Figures 2 and 3. For use, a thin layer of ultrasound gel is applied to the probe before positioning a protective finger covering. A second layer of sterile gel, or alternatively an antiseptic product, is then applied externally to the sheath before contact with the skin. During scanning, gentle and even pressure must be maintained to avoid vascular compression. Between patients, the probe is disinfected with a compatible low-level disinfectant.

To guarantee real-time stability, the SibUS-In uses a wired connection, avoiding signal lag or battery issues common with wireless devices. Although the device uses a wired connection, it remains fully compatible with wireless touchscreens, enhancing flexibility and workflow efficiency in diverse clinical settings. At present, certain advanced functionalities, such as automated needle enhancement and tracking, are not yet implemented in this generation of the device.

### Applications

The SibUS-In probe can be integrated into 4 key procedural stages, making ultrasound guidance a continuous and seamless process: preinjection mapping, injection, immediate postinjection, and postprocedural management. In the preinjection phase, it enables real-time vascular mapping and the identification of residual fillers or implanted materials, enhancing safety and procedural planning. During the injection phase, continuous ultrasound monitoring ensures precise and consistent needle or cannula placement and real-time visualization of filler flow, helping to mitigate the risk of vascular injury or product misplacement. In the immediate postinjection phase, it allows evaluation of filler distribution and early detection of complications, including vascular occlusion or hematoma. Finally, in the postprocedural phase, the probe serves as both a diagnostic and therapeutic tool for managing complications, facilitating targeted interventions and improving patient outcomes.

Unlike conventional ultrasound probes that interrupt workflow or require a second operator, SibUS-In maintains the injector's natural 2-handed technique. The injector's dominant hand manipulates the syringe, whereas the noninjecting hand controls the fingertip probe and maintains skin palpation, enabling a fluid, uninterrupted workflow. In practice, this configuration allows rapid alternation between scanning and injecting without repositioning or external assistance.

**Figure 3.**
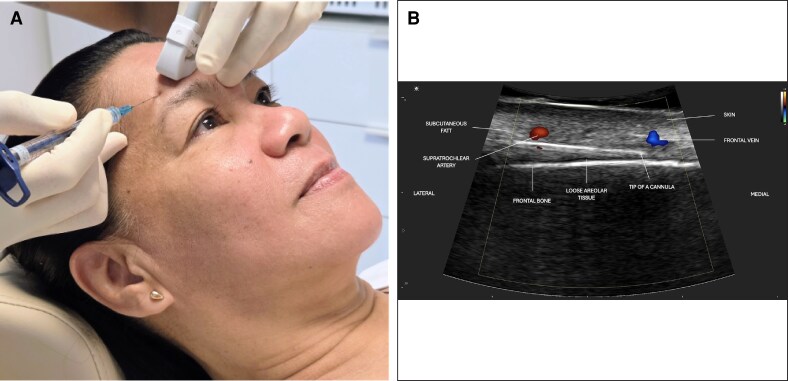
In a 55-year-old female patient, (A) clinical view shows ultrasound-guided filler injection in the forehead with the SIBUS-In probe using a 25 G cannula. (B) The corresponding Doppler ultrasound image demonstrates the cannula fully visualized in-plane, with the supratrochlear artery and frontal vein clearly identified, allowing safe navigation and precise filler placement.

**Figure 4.**
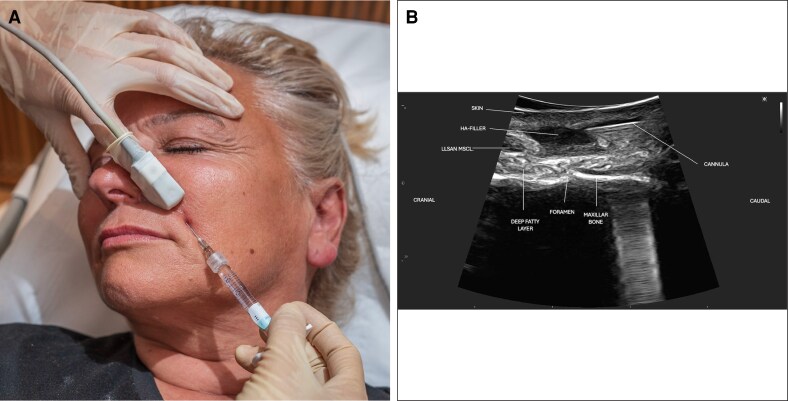
In a 59-year-old female patient, (A) clinical view shows ultrasound-guided hyaluronic acid injection into the nasolabial fold using the SIBUS-In probe. (B) The corresponding grayscale ultrasound image demonstrates the hyaluronic acid filler as hypoechoic material along the tip of a 25 G cannula, fully visualized with the in-plane technique.

Table 1 provides an overview of the main challenges encountered in specific facial regions and highlights the corresponding SibUS-In solutions developed to address them. Figures 3 and 4 illustrate how the SibUS-In probe is positioned during injections and how the corresponding ultrasound views integrate into the procedural workflow. Additional examples of its use across multiple facial regions—including both botulinum toxin and filler applications—are presented in Supplemental Figures 4-17, which provide paired clinical views and ultrasound images for each zone. A full demonstration of probe handling and synchronized real-time imaging is available in the Video.

**Table 1.** Injection Challenges and Proposed SIBUS Solutions by Facial Zone

Challenge type	Facial zones affected	Key anatomical structures	SIBUS solutions
Vascular risks	All zones	Supratrochlear a., supraorbital a., superficial temporal a., frontal branch of superficial temporal a., deep temporal a., zygomatico-orbital a., infraorbital a., angular a., transverse facial a., superior labial a., inferior labial a., facial a., dorsal nasal a., lateral nasal a., columellar a., submental a., mental a., ascending mental a.	Real-time Doppler imaging enables vascular mapping, reduces the risk of arterial or venous occlusion, and prevents compression through precise depth control.
Asymmetry or uneven distribution	All zones	Corrugator m., procerus m., frontalis m., orbicularis oculi m., zygomaticus m., levator labii superioris alaeque nasi m., levator labii superioris m., masseter m., orbicularis oris m./modiolus m., depressor anguli oris m., mentalis m.	High-resolution imaging of facial muscles supports accurate filler and toxin placement, optimizing intramuscular targeting and uniform distribution.
Filler overcorrection	Periorbital and perioral	SOOF and ROOF fat compartments, orbicularis oculi m., orbicularis oris m.	Real-time feedback during injections helps prevent overcorrection by visualizing filler deposition in real time.
Difficult anatomy	Glabella, forehead, temple, periorbital region, perioral region, lateral cheek, nasal	Frontalis m., corrugator m., interfascial space in the temple, SOOF and ROOF fat compartments, parotid gland, masseter m., nasal bones, and cartilage	Curved ergonomic design combined with high-frequency imaging improves navigation in complex zones, differentiates anatomical layers, and facilitates precise, low-risk product placement with minimal surface irregularities.
Patient comfort	All zones	N/A	Minimal gel application designed to reduce patient discomfort.
Minimizing operator fatigue and procedural time	All zones	N/A	Fingertip ergonomic design and lightweight construction decrease operator strain and improve precision during prolonged procedures.
Dynamic navigation	All zones	N/A	Fingertip integration allows natural, dynamic adjustments during injections, enhancing control and aiming to improve procedural safety through enhanced visualization of vascular structures.

a.: artery; m.: muscle; N/A, not applicable.

### Comparison with Traditional Probes

Conventional linear or hockey-stick probes were originally designed for radiology or emergency medicine rather than facial injections. They are rigid and cumbersome, require large amounts of ultrasound gel, and offer limited maneuverability in curved or narrow facial areas. Their use often confines the practitioner to a single-handed workflow, increasing operator fatigue and interrupting procedural flow.

In contrast, the SibUS-In probe was developed specifically for facial aesthetics. In our experience, it provides high-definition imaging comparable in clarity to larger cart-based systems while remaining lightweight and ergonomically balanced. The optimized frequency range (from 7 to 15 MHz) combines excellent superficial resolution with sufficient penetration for deeper structures.

Supplemental Figures 18 and 19 present comparative imaging obtained with a high-end cart-based system (LOGIQ P9, 15 MHz; GE Healthcare, Chicago, IL), a mid-range system (VENUE Fit, 20 MHz; GE Healthcare), a handheld wireless device (Vscan Air, 12 MHz; Clarius, Burnaby, Canada), and the SibUS-In probe (15 MHz). These comparisons illustrate that, although the SibUS-In's 15 MHz bandwidth does not reach the theoretical resolution of optimized 20 MHz probes, the resulting image quality appears sufficiently detailed for facial soft-tissue and vascular assessment. The fingertip probe therefore achieves a practical balance between resolution, penetration, and ergonomic control, making it suitable for real-time injection monitoring.

Its fingertip placement offers improved control and facilitates access to small or curved facial regions, such as the tear trough, temple, and pyriform space, areas that can be challenging to approach with conventional probes. In our clinical use, this configuration aligns naturally with the injector's hand motion and enhances maneuverability in confined anatomical zones. In addition, the fingertip-mounted configuration reduces operator fatigue, supporting longer and more precise procedures without loss of comfort.

According to our experience, the SibUS-In probe helps mitigate several limitations encountered with traditional ultrasound devices. Table 2 summarizes these contrasts in terms of precision, usability, and comfort, highlighting that SibUS-In is not merely a smaller probe, but one conceived to integrate naturally into the injector's workflow.

**Table 2.** Comparison of SibUS-In With Traditional and Portable Ultrasound Probes

Feature	Linear probes	Hockey-stick probes	SIBUS-In probe
Frequency	7-15 MHz	10-14 MHz	7-15 MHz
Resolution (superficial layers)	Good	Good	Good
Penetration depth	Moderate	Moderate	Moderate
Real-time feedback	Real-time visualization	Real-time visualization	Real-time visualization
Anatomical navigation	Limited on curved surfaces	Improved flexibility	Optimized for curved and small facial zones but limited field of view compared with larger probes.
Ergonomics	Bulky, requires 1-hand operation	Smaller, still requires 1-hand operation	Fingertip integration enables 2-hand operation and improved dexterity; a slight adaptation needed for first-time users.
Workflow integration	May disrupt procedural flow	Partial integration	Seamless integration into standard injection workflow
Gel application	High-gel requirement	Moderate gel requirement	Minimal gel requirement
User fatigue and procedural time	High during prolonged use	Moderate	Reduced because of lightweight form

In our experience, the transition from blind to ultrasound-guided injections using SibUS-In is smooth and intuitive, requiring minimal adaptation. Compared with conventional ultrasound systems, it allows a more ergonomic and fluid workflow, as both hands remain engaged throughout the injection process, an observation consistently reported during our clinical use.

## DISCUSSION

Ultrasound has become an increasingly valuable adjunct in aesthetic medicine, offering real-time visualization of vascular and soft-tissue structures that can enhance both safety and precision.^3,6-8^ Severe complications of aesthetic injections such as vascular occlusion, skin necrosis, visual loss, and inflammatory reactions remain rare but potentially devastating. Literature reviews highlight a concerning rise in reported blindness cases: 48 cases were published between January 2015 and September 2018, and 365 cases between September 2018 and March 2023. Alarmingly, almost 70% of treated patients did not regain vision.^9,10^ These data emphasize the importance of vascular awareness during injection procedures.

Traditional safety techniques, such as aspiration before injection, slow injection, or the use of blunt-tip cannulas, provide only partial protection, because they depend entirely on operator skill and cannot account for anatomic variability.^11-14^ In contrast, ultrasound enables direct visualization of vessels and soft tissues, allowing dynamic guidance of the needle or cannula and helping to mitigate these risks.

Accurate product placement represents another major advantage of real-time imaging. Misplacement into the superficial muscular aponeurotic system or parotid gland remains a known source of nodules, asymmetries, and contour irregularities.^15,16^ Continuous ultrasound visualization allows injectors to verify needle depth and plane in real time, thereby reducing the risk of unintentional injection or product misdistribution.

Beyond prevention and improved placement accuracy, ultrasound has also demonstrated value in complication management.^17^ Schelke et al reported that ultrasound-guided hyaluronidase injections required significantly lower doses (95 IU) than flooding protocols (1519 IU) while achieving faster and more complete resolution.^4^ These findings highlight how ultrasound can enhance both the safety and therapeutic precision of injectable procedures.

Despite these advantages, ultrasound has historically remained underutilized in aesthetic practice because of the ergonomic and technical limitations of conventional probes. Devices originally designed for radiology or emergency medicine are often bulky, cumbersome, and linear and are not suited to the precise manual control required for facial injections or to the curved contours of the face, frequently requiring a second operator and disrupting workflow.

The SibUS-In system was specifically engineered to address these limitations. Its fingertip-mounted, lightweight, and curved design integrates imaging directly into the injector's natural workflow, allowing the practitioner to palpate, scan, and inject simultaneously while maintaining a natural, 2-handed technique. Early clinical experience suggests that the integration of Doppler imaging at the injector's fingertip facilitates continuous vascular monitoring and enhances procedural control without interrupting workflow. This dual-hand coordination not only supports targeted complication management and consistent, precise filler placement but may also help reduce vascular risk by allowing continuous real-time control during injection.

The applicability of SibUS-In extends beyond facial aesthetics. In plastic surgery, ultrasound is already used for pectoralis nerve blocks before breast and anterior chest surgery, augmentation mammaplasty, breast and gluteal fat grafting, filler injections in the buttocks, ultrasound-guided rectus abdominis fat transfer, and vascularized flap harvesting. Where conventional probes often require 2-operator setup, the SibUS-In configuration enables single-operator imaging and intervention, improving procedural autonomy and efficiency.

Although training and equipment costs may initially limit adoption, the intuitive handling of SibUS-In shortens the learning curve compared with conventional systems. Leasing models and dedicated training programs may further support adoption, particularly among smaller or newly equipped practices.

From a regulatory perspective, ultrasound guidance is rapidly becoming an expected safety standard. In gluteal fat grafting (eg, the Brazilian butt lift), for example, verification of subcutaneous injection under ultrasound is now mandatory in several jurisdictions.^18^ In Florida, timestamped ultrasound recordings are legally required in the patient's medical file, reflecting the direction of policy change based on recommendations from academic societies.^19^ It is foreseeable that similar safety frameworks will eventually extend to fillers and neuromodulators, reinforcing the need to integrate ultrasound education into aesthetic and surgical curricula.

Collectively, these developments position SibUS-In as a practical bridge between ultrasound's established safety benefits and its daily clinical application. Although current evidence remains preliminary and largely experiential, the system's ergonomic design and seamless workflow integration address the practical barriers that have historically limited the routine use of ultrasound in aesthetics.

### Limitations

Although the SibUS-In probe represents an important step toward practical ultrasound-guided injections, several limitations should be acknowledged. Effective use requires foundational ultrasound training, including orientation, Doppler interpretation, and real-time coordination of the probe and needle. Although ergonomically designed to fit the index finger, the digit conventionally used for palpation, the combined use of tactile feedback and imaging may initially feel unfamiliar to some operators. However, compared with conventional ultrasound-guided injections that require mastering single-handed techniques while holding a bulky probe, SibUS-In tends to be easier to handle and less fatiguing to use.

Beyond ergonomics, image quality also has inherent limitations. The optimized 15 MHz frequency range offers excellent definition of facial structures but does not yet achieve the microscopic detail provided by optimized 20 MHz systems. Despite its high-resolution imaging, consistent visualization of very fine needle tips (32, 30, or 27 G) in deeper tissue layers remains challenging, introducing a degree of placement uncertainty. This underscores the importance of echogenic, pretreated needles, now available in 27 and 30 G sizes and lengths of 13 and 20 mm, as well as echogenic cannulas in 22 to 27 G.

From a functional standpoint, the current generation does not yet include certain advanced features found in high-end ultrasound platforms, such as automated needle enhancement or artificial intelligence (AI)-assisted vascular mapping. Although such tools may offer added convenience, they are not essential for the effective clinical use of SibUS-In, whose core functionality already supports precise, real-time injection guidance.

In terms of workflow integration, setup and handling remain key practical considerations. Although the use of ultrasound gel and a wired connection may slightly extend preparation time compared with blind injections, these requirements are similar to those of other portable ultrasound systems. A workspace organized to minimize cable interference and facilitate movement further enhances procedural fluidity. For practitioners already accustomed to ultrasound, overall setup time remains comparable.

Cost and accessibility are additional considerations when comparing ultrasound systems. Detailed economic analyses were beyond the scope of this study, but future work could evaluate cost–benefit aspects relative to other devices to better inform clinical adoption.

Looking ahead, continued operator training and technological refinement are expected to gradually minimize these limitations and support broader clinical adoption. Ultimately, the technique mirrors standard injection practices but adds 1 transformative capability: the injector can visualize the needle's position in real time. Finally, the absence of quantitative real-world data and performance metrics remains a current limitation. Further studies are warranted to evaluate safety outcomes, learning curves, reproducibility, and procedural efficiency using standardized protocols.

### Future Directions

Further research will be required to quantitatively evaluate efficacy, reproducibility, and interoperator reliability under standardized conditions. To this end, multicenter validation studies are currently being designed to assess reproducibility and the consistency of imaging interpretation across key facial regions commonly treated in aesthetic practice, including the temple, nasolabial fold, and periorbital area. These investigations aim to provide the quantitative evidence necessary to support broader clinical validation and long-term adoption.

Also, future improvements will likely include the integration of secure, high-performance wireless technology, eliminating cable constraints while maintaining image reliability. Achieving this goal will require further refinement of wireless platforms, because current ultrasound technologies, across all manufacturers, have not yet reached the reliability standards essential for high-stakes clinical use.

Advances in echogenic materials and software-based tip tracking will further enhance visualization of fine needles and cannulas. AI represents another promising frontier: real-time anatomical layer recognition, color-coded vascular mapping, and predictive risk detection could guide injectors step by step. Machine learning could also refine Doppler signal interpretation and early complication alerts.

Looking ahead, next-generation platforms could incorporate gesture or voice control for sterile, hands-free manipulation and integrate AI algorithms for tailored treatment recommendations. Because SibUS-In aligns with the natural workflow of injectors, it provides an ideal ergonomic foundation for the integration of these forthcoming innovations.

## CONCLUSIONS

The SibUS-In system introduces a new ergonomic format for ultrasound-guided aesthetic injections, integrating high-resolution imaging onto the injector's fingertip. This configuration enables simultaneous palpation, scanning, and injection, restoring full manual control and addressing one of the main barriers that have limited ultrasound adoption in aesthetic practice. By adapting ultrasound to the injector's natural workflow, the device facilitates real-time vascular visualization and more precise product placement without compromising dexterity. Although quantitative data are still needed to confirm its clinical benefits, early experience suggests that this fingertip-mounted design represents a significant step toward making image-guided injections safer, more intuitive, and more widely accessible in aesthetic medicine.

## Supplemental Material

This article contains supplemental material located online at https://doi.org/10.1093/asjof/ojag046.

## Supplementary Material

ojag046_Supplementary_Data
