# Supplementary material for: The SibUS-In Finger Probe: An Alternative Device and Method for Ultrasound-Guided Injections
Source: Aesthet Surg J Open Forum. 2026 Mar 25;8:ojag046. doi: 10.1093/asjof/ojag046 (PMC13098122; doi:10.1093/asjof/ojag046)
Supplement: ojag046_Supplementary_Data [file ojag046_Supplementary_Data.zip › Supplemental Figure Legend.docx]

**Supplemental Figure Legend**

**Supplemental Figure 1.** SIBUS-In® system mounted on a mobile trolley for clinical use.

**Supplemental Figure 2.** Probe positioning on the index finger of the non-injecting hand using the SibUS-In® fingertip transducer, providing stable contact, minimal pressure on the tissue, and real-time visualization during injection.

**Supplemental Figure 3.** Preparation for a hyaluronic acid injection with the simultaneous use of the SIBUS-In fingertip-mounted ultrasound probe, allowing real-time ultrasound guidance while maintaining a natural injection workflow and control of both needle and imaging.

**Supplemental Figure 4.** Ultrasound-guided botulinum toxin injection into the corrugator muscle using the SibUS-In® probe in a 35-year-old male patient: (A) clinical view showing probe placement and injection; (B) corresponding gray-scale ultrasound image demonstrating in-plane needle visualization during toxin delivery into the corrugator muscle.

**Supplemental Figure 5A–B.** Ultrasound-guided botulinum toxin injection into the frontalis muscle using the SibUS-In® probe in a 35-year-old male patient: (A) clinical view illustrating probe placement and injection technique for forehead wrinkle relaxation; (B) corresponding gray-scale ultrasound image showing out-of-plane needle visualization during toxin delivery into the frontalis muscle.

**Supplemental Figure 6A–B.** Tear trough vascular mapping using the SibUS-In® probe in a 59-year-old female patient: (A) clinical view showing the probe positioned obliquely along the tear trough region for vascular mapping; (B) corresponding color Doppler ultrasound image demonstrating the angular vein with the probe in the same oblique orientation.

**Supplemental Figure 7A–B.** Ultrasound-guided botulinum toxin injection in the periorbital region using the SibUS-In® probe in a 55-year-old female patient: (A) clinical view showing probe positioning and needle orientation toward the orbicularis oculi muscle; (B) corresponding gray-scale ultrasound image demonstrating out-of-plane needle visualization for toxin delivery into the orbicularis oculi muscle.

**Supplemental Figure 8A–B.** Ultrasound-guided hyaluronic acid injection into the malar region using the SibUS-In® probe in a 59-year-old female patient: (A) clinical view showing probe placement and cannula approach; (B) Gray-scale ultrasound image demonstrating the 25G cannula tip fully visualized along its length within the deep malar fat pad using the in-plane technique.

**Supplemental Figure 9A–B.** Nasal dorsum vascular mapping using the SibUS-In® probe in a 59-year-old female patient: (A) clinical view showing the probe positioned vertically on the nasal dorsum at the transition between the nasal bone and the upper lateral cartilage; (B) corresponding color Doppler ultrasound image demonstrating the dorsal nasal artery.

**Supplemental Figure 10A–B.** Ultrasound assessment of the nasolabial region using the SibUS-In® probe in a 55-year-old female patient: (A) clinical view showing the probe positioned along the lateral aspect of the nose; (B) corresponding gray-scale ultrasound image demonstrating the levator labii superioris alaeque nasi (LLSAN) muscle visualized along its length and attached to the upper frontal process of the maxilla.

**Supplemental Figure 11A–B.** Lower lip vascular mapping using the SibUS-In® probe in a 55-year-old female patient: (A) clinical view showing the probe positioned horizontally on the lower lip; (B) corresponding color Doppler ultrasound image demonstrating the inferior labial artery in relation to the orbicularis oris muscle, labial salivary gland, and oral mucosa.

**Supplemental Figure 12A–B.** Ultrasound-guided depressor anguli oris (DAO) neurotoxin procedure using the SibUS-In® probe in a 55-year-old female patient: (A) clinical view showing probe positioning and injection approach to the DAO region; (B) corresponding gray-scale ultrasound image demonstrating in-plane visualization of the needle (red arrow) approaching the DAO muscle.

**Supplemental Figure 13A–B.** Ultrasound assessment of the masseter region using the SibUS-In® probe in a 59-year-old female patient: (A) clinical view showing the probe positioned horizontally over the mandibular region; (B) corresponding gray-scale ultrasound image demonstrating the masseter muscle and its deep inferior tendon (DIT) separating the superficial and deep bellies.

**Supplemental Figure 14A–B.** Ultrasound-guided mentalis neurotoxin procedure using the SibUS-In® probe in a 55-year-old female patient: (A) clinical view showing probe positioning and injection approach to the mentalis region; (B) corresponding gray-scale ultrasound image demonstrating in-plane visualization of the needle (red arrow) approaching the mentalis muscle.

**Supplemental Figure 15A–B.** Ultrasound-guided masseter neurotoxin procedure using the SibUS-In® probe in a 55-year-old female patient: (A) clinical view showing probe positioning over the masseter region; (B) corresponding gray-scale ultrasound image showing full-length, in-plane visualization of the needle (red arrow) within the masseter muscle.

**Supplemental Figure 16A–B.** Ultrasound-guided jawline hyaluronic acid procedure using the SibUS-In® probe in a 59-year-old female patient: (A) clinical view showing probe positioning and 25G cannula approach along the jawline using the in-plane technique; (B) corresponding gray-scale ultrasound image showing full-length, in-plane visualization of the cannula positioned within the superficial fatty layer, in close relation to the parotid gland.

**Supplemental Figure 17A–B.** Ultrasound-guided marionette line hyaluronic acid procedure using the SibUS-In® probe in a 59-year-old female patient: (A) clinical view showing oblique probe positioning and 25G cannula approach along the marionette line; (B) corresponding gray-scale ultrasound image showing in-plane visualization of the cannula positioned within the superficial fatty layer, in close relation to the modiolus and the depressor anguli oris muscle.

**Supplemental Figure 18.** B-mode imaging of the deep pyriform space using four different probes, from left to right: high-end LOGIQ P9 (GE Healthcare, 15 MHz), mid-range VENUE Fit (GE Healthcare, 20 MHz), handheld Vscan Air (Clarius, 12 MHz), and fingertip-mounted SIBUS-In (15 MHz).

**Supplemental Figure 19.** Color Doppler imaging of the deep pyriform space with the same four probes, from left to right: high-end LOGIQ P9 (GE Healthcare, 15 MHz), mid-range VENUE Fit (GE Healthcare, 20 MHz), handheld Vscan Air (Clarius, 12 MHz), and fingertip-mounted SIBUS-In (15 MHz).
