# Supplementary figures and images for: The SibUS-In Finger Probe: An Alternative Device and Method for Ultrasound-Guided Injections
Source: Aesthet Surg J Open Forum. 2026 Mar 25;8:ojag046. doi: 10.1093/asjof/ojag046 (PMC13098122; doi:10.1093/asjof/ojag046)

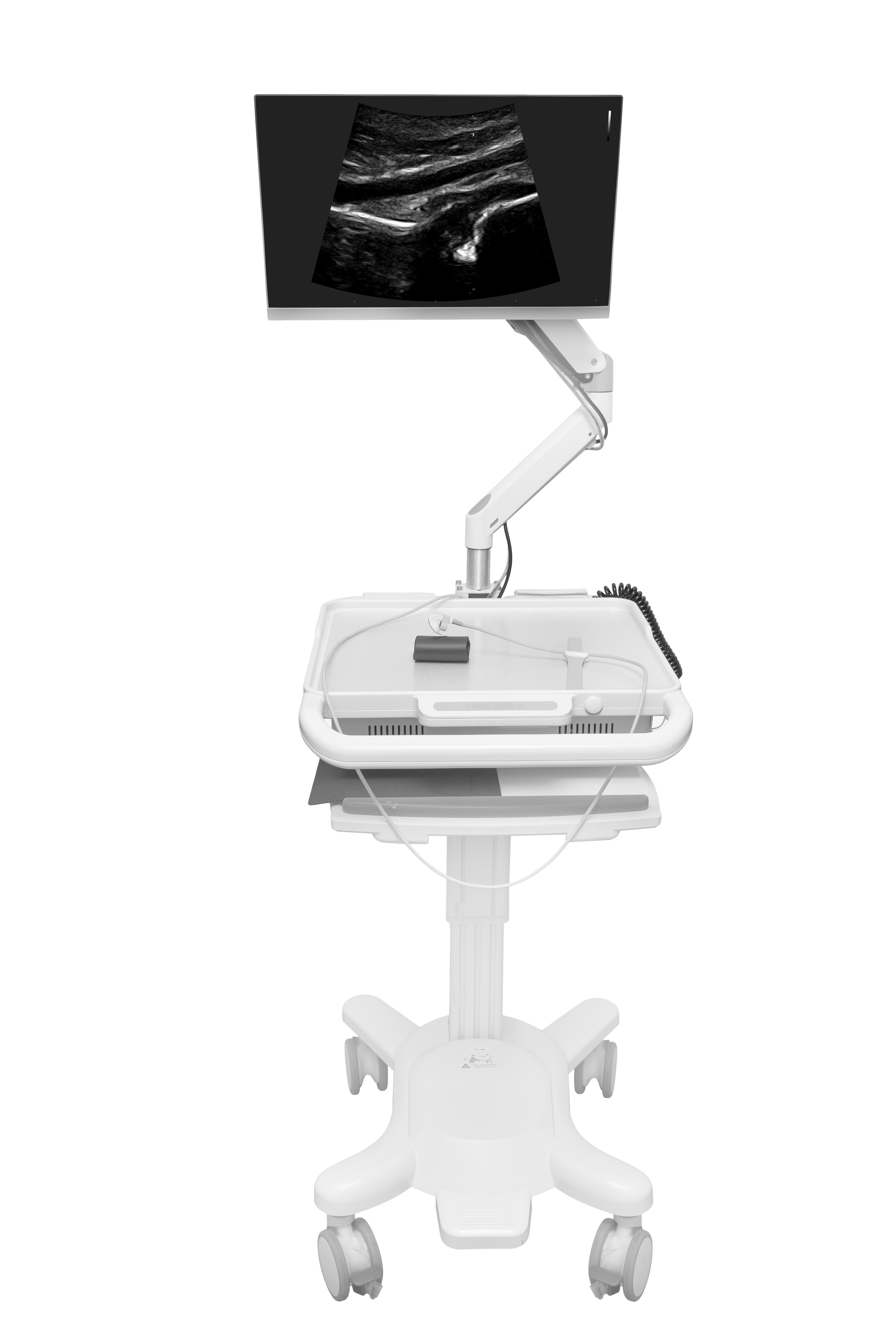

Supplement: ojag046_Supplementary_Data [file ojag046_Supplementary_Data.zip › Supplemental Figure 1.png]

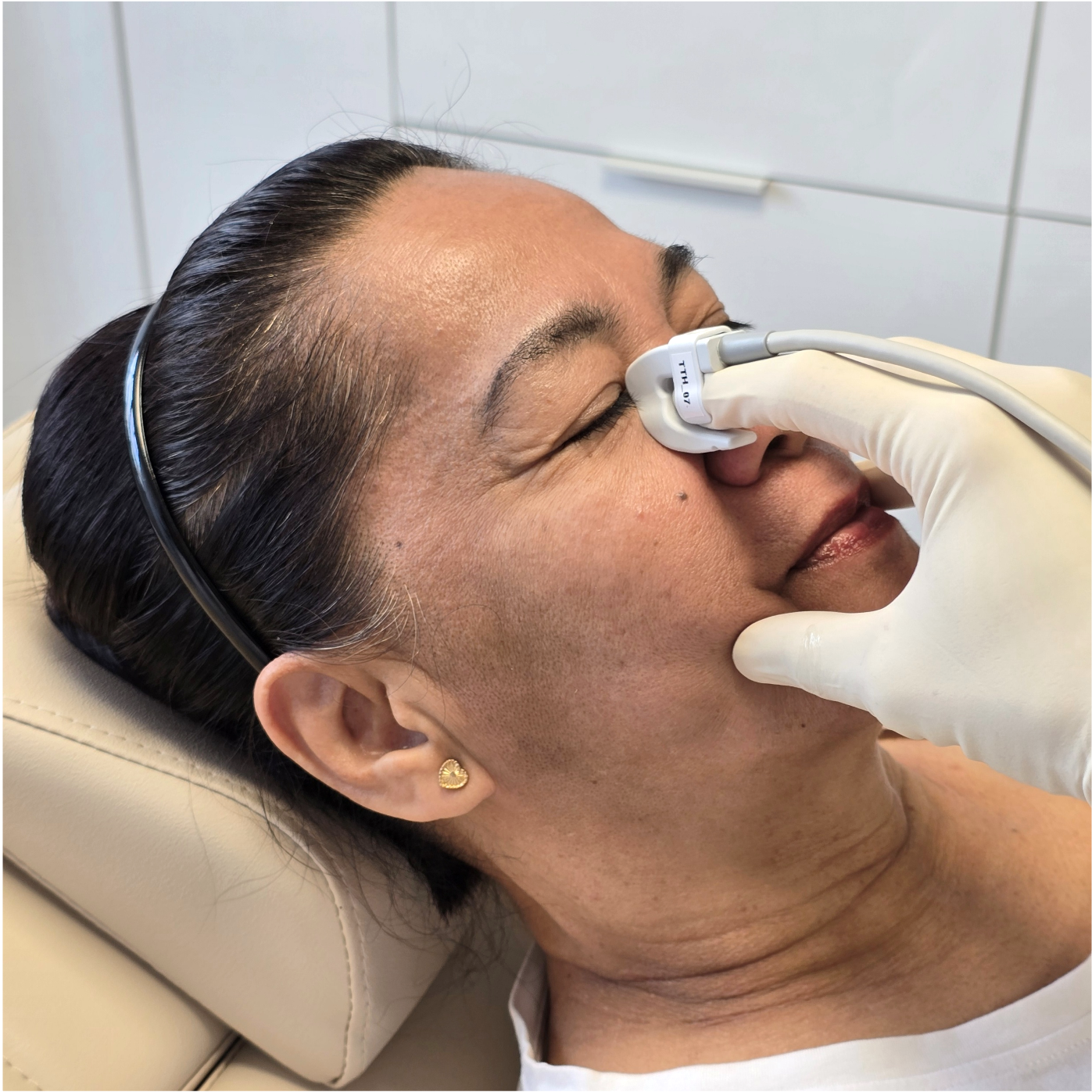

Supplement: ojag046_Supplementary_Data [file ojag046_Supplementary_Data.zip › Supplemental Figure 10A.png]

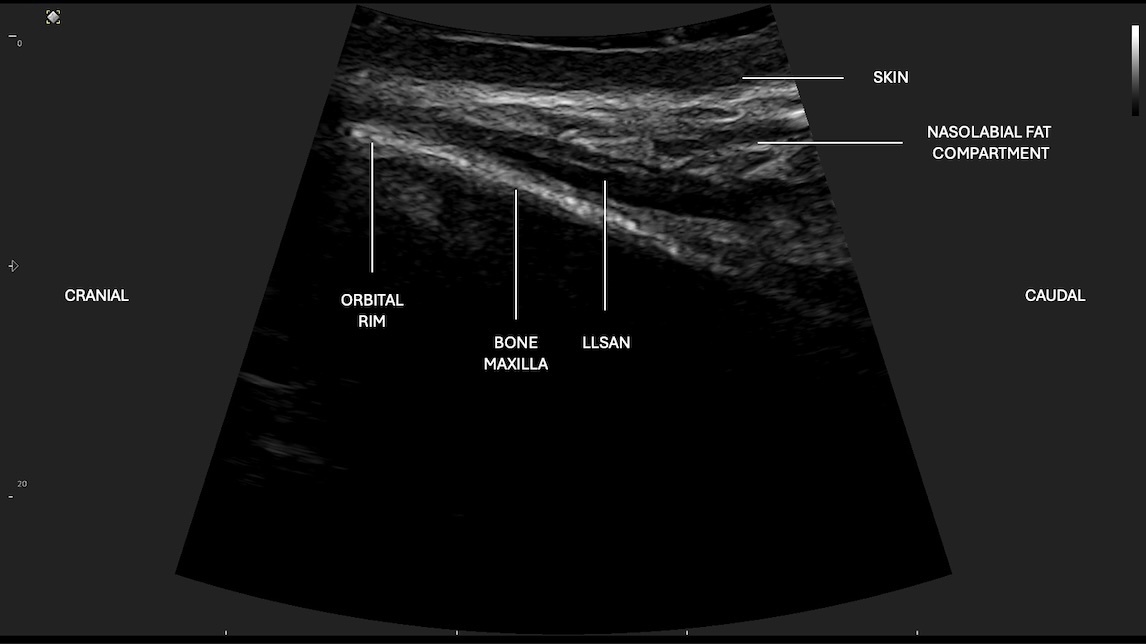

Supplement: ojag046_Supplementary_Data [file ojag046_Supplementary_Data.zip › Supplemental Figure 10B.jpg]

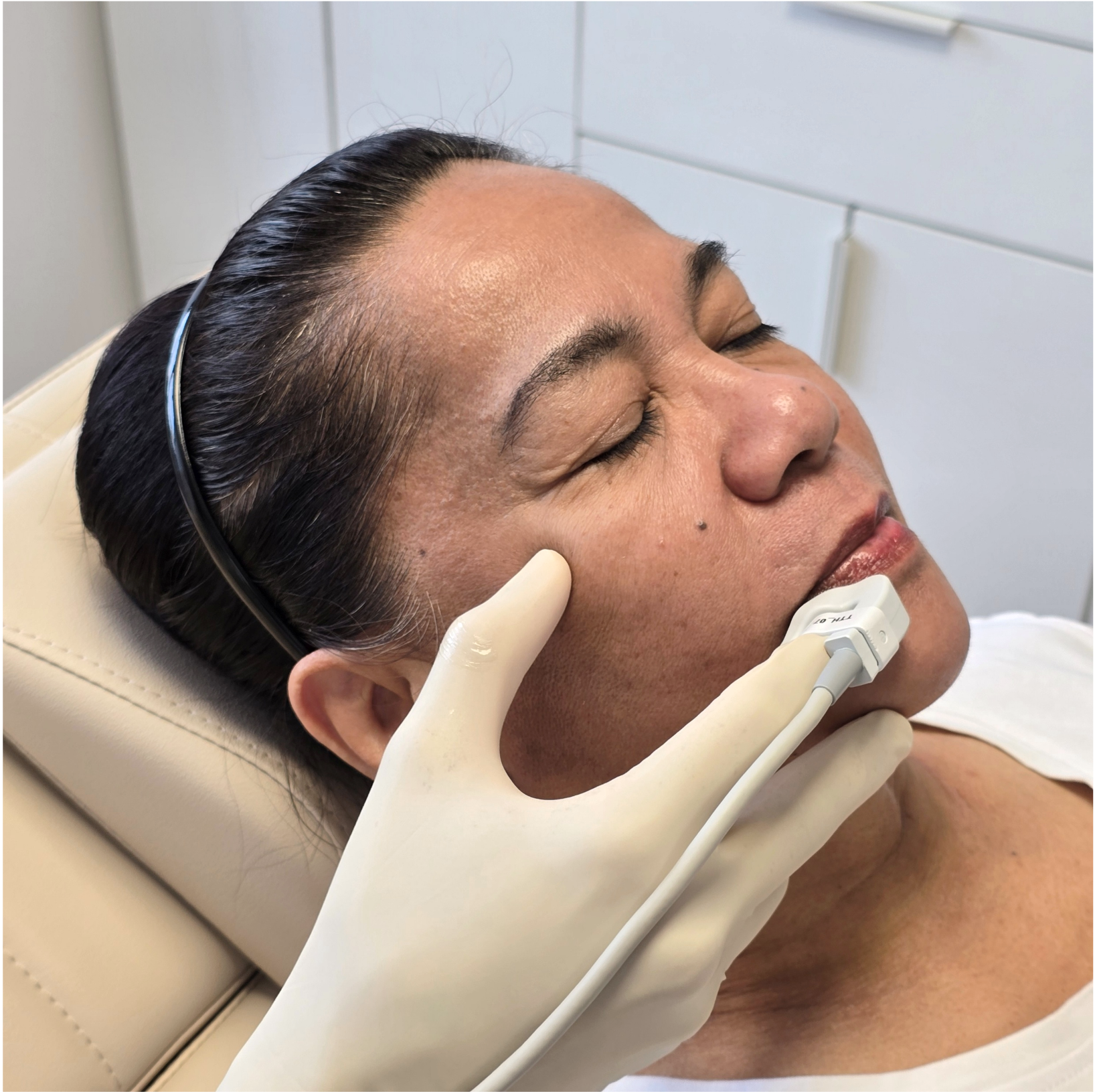

Supplement: ojag046_Supplementary_Data [file ojag046_Supplementary_Data.zip › Supplemental Figure 11A.png]

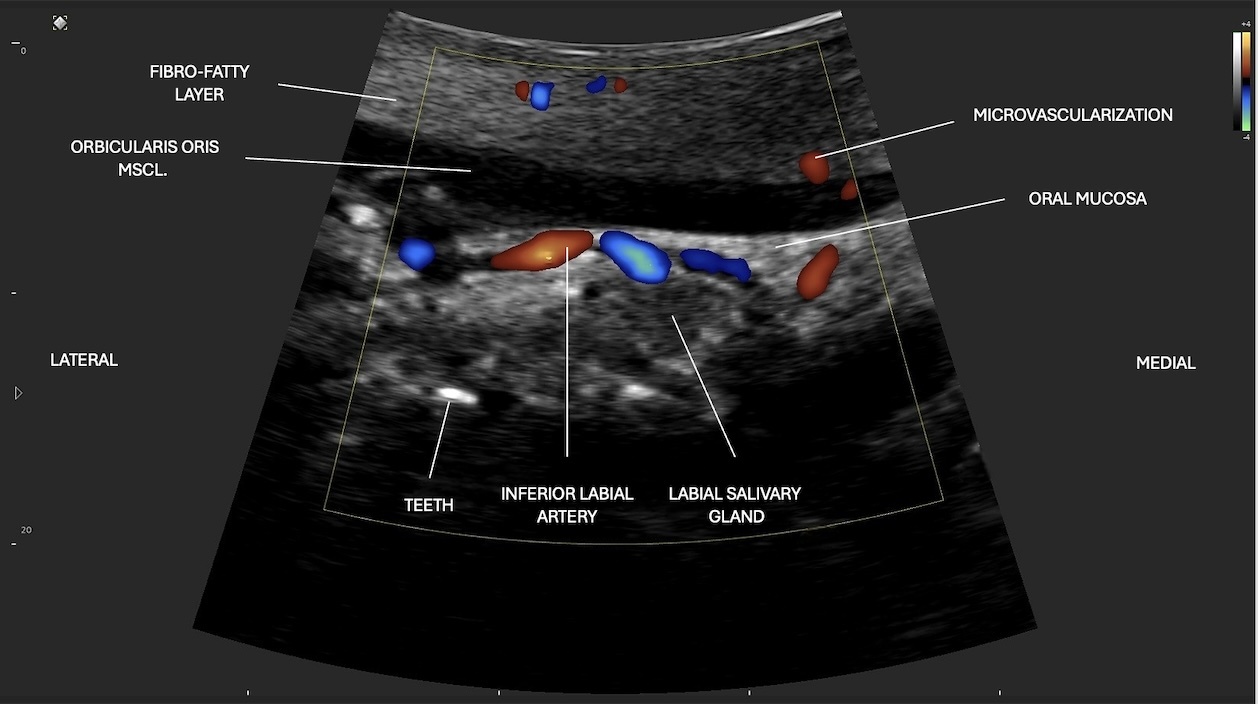

Supplement: ojag046_Supplementary_Data [file ojag046_Supplementary_Data.zip › Supplemental Figure 11B.jpg]

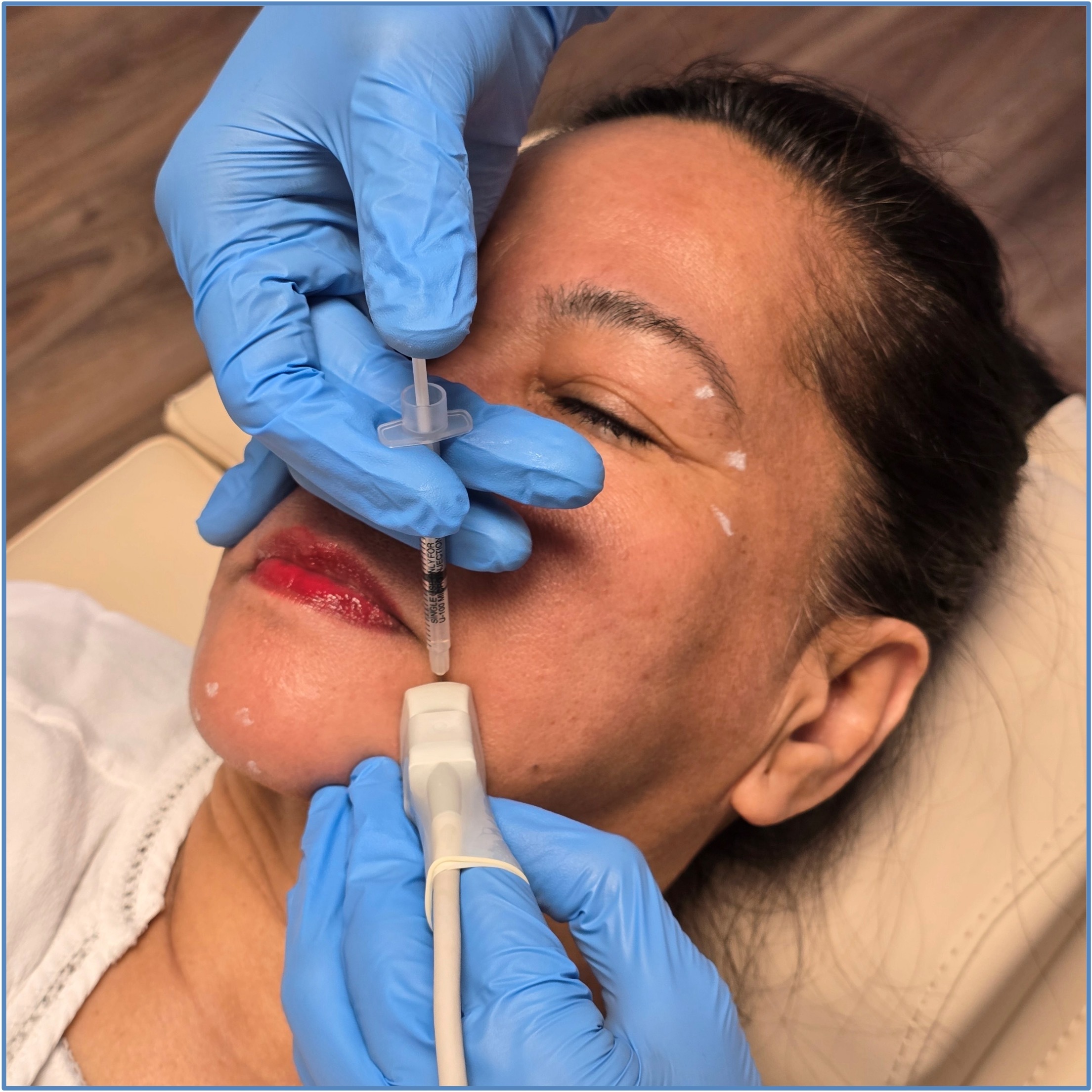

Supplement: ojag046_Supplementary_Data [file ojag046_Supplementary_Data.zip › Supplemental Figure 12A.jpg]

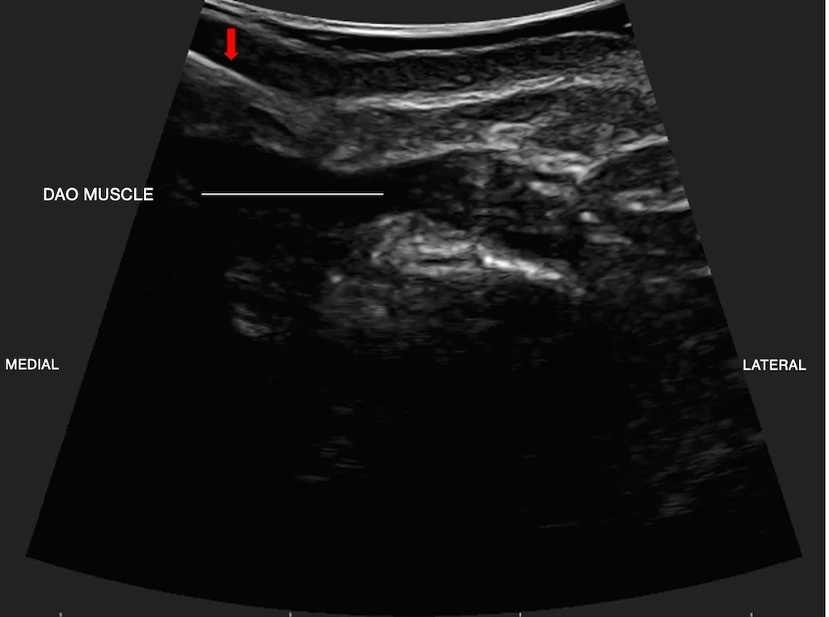

Supplement: ojag046_Supplementary_Data [file ojag046_Supplementary_Data.zip › Supplemental Figure 12B.jpg]

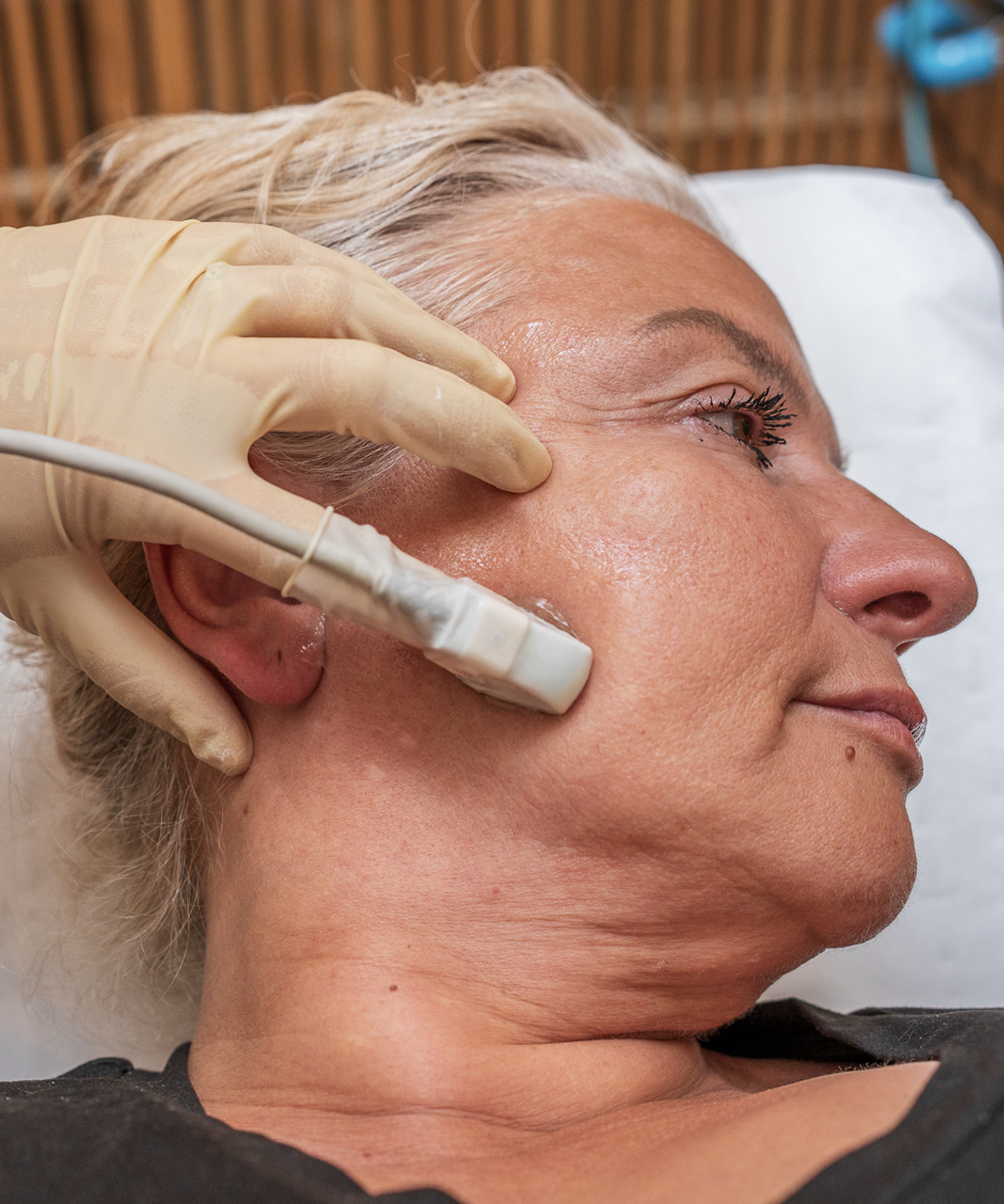

Supplement: ojag046_Supplementary_Data [file ojag046_Supplementary_Data.zip › Supplemental Figure 13A.jpg]

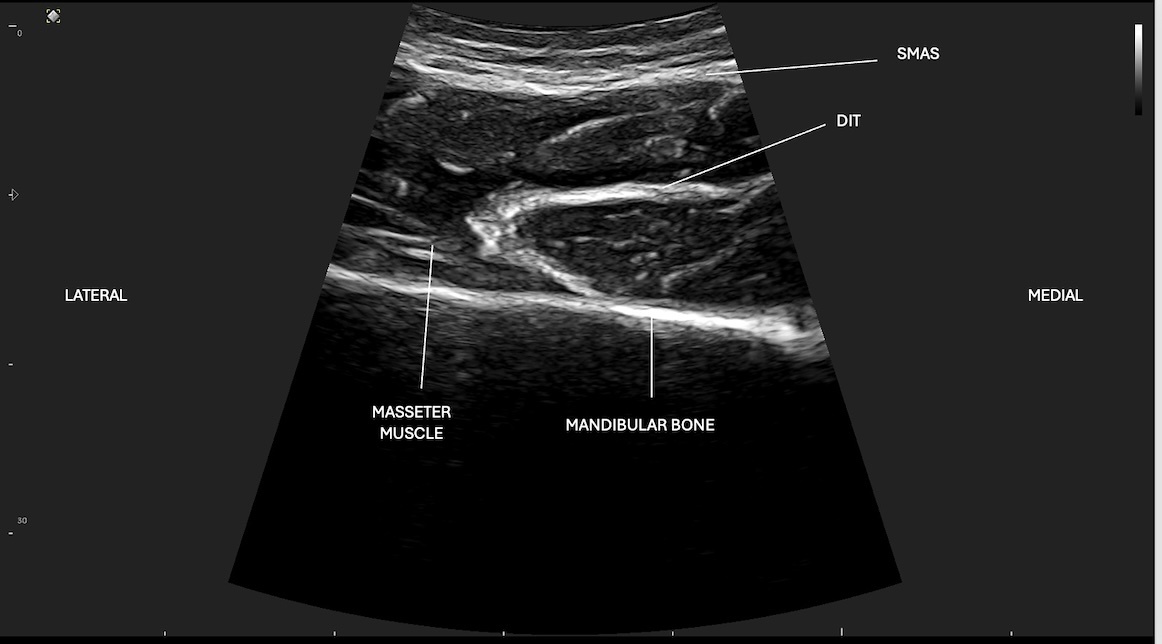

Supplement: ojag046_Supplementary_Data [file ojag046_Supplementary_Data.zip › Supplemental Figure 13B.jpg]

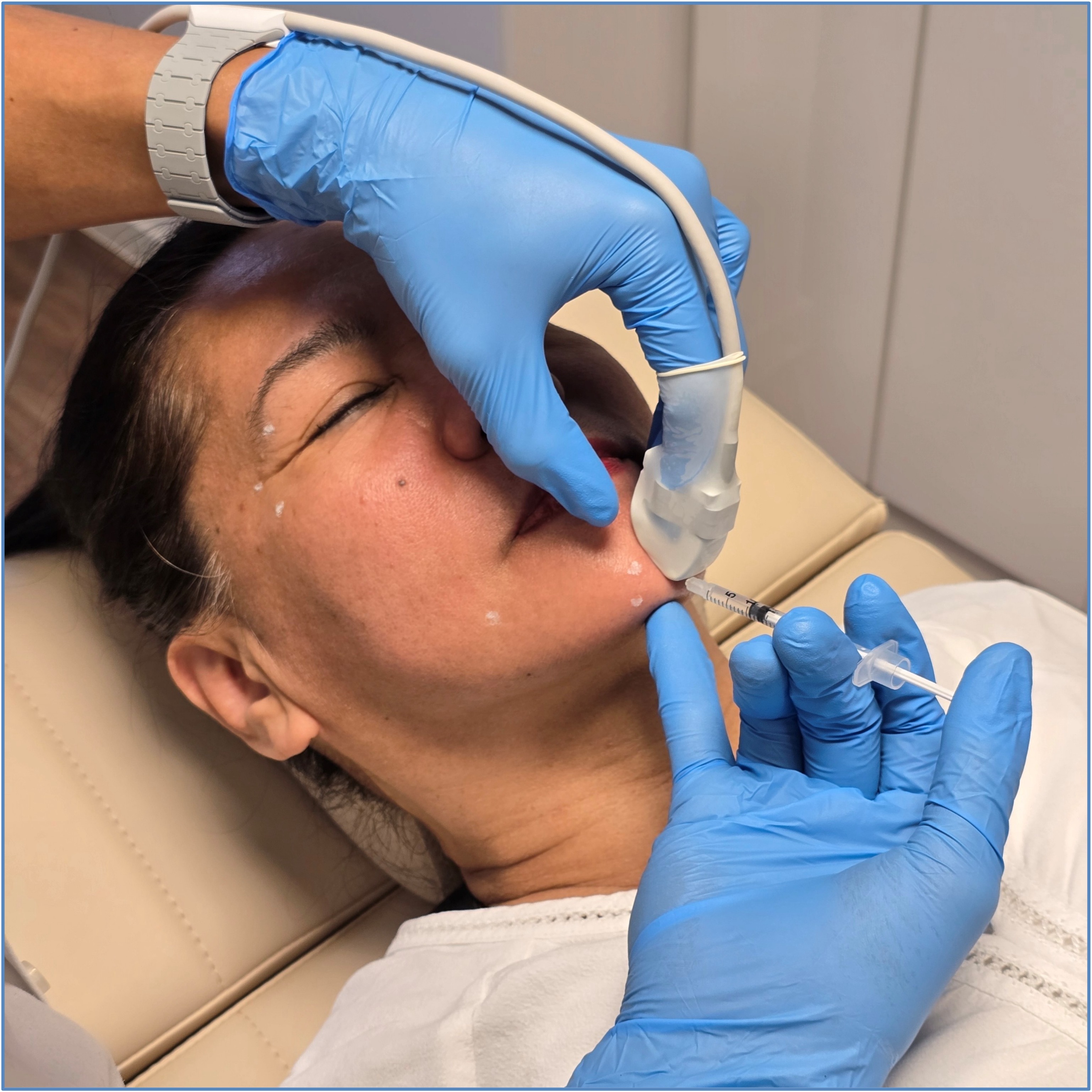

Supplement: ojag046_Supplementary_Data [file ojag046_Supplementary_Data.zip › Supplemental Figure 14A.jpg]

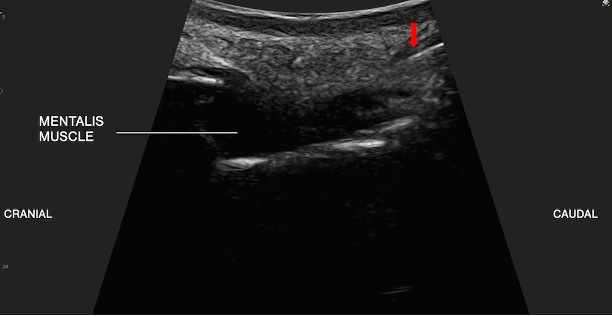

Supplement: ojag046_Supplementary_Data [file ojag046_Supplementary_Data.zip › Supplemental Figure 14B.jpg]

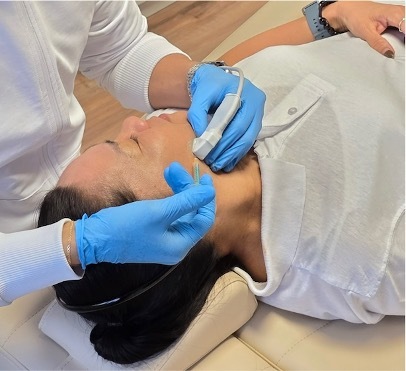

Supplement: ojag046_Supplementary_Data [file ojag046_Supplementary_Data.zip › Supplemental Figure 15A.jpg]

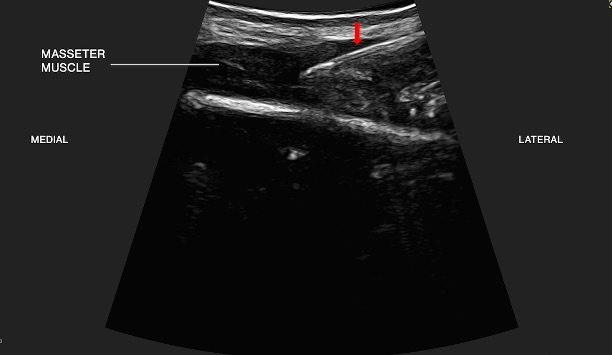

Supplement: ojag046_Supplementary_Data [file ojag046_Supplementary_Data.zip › Supplemental Figure 15B.jpg]

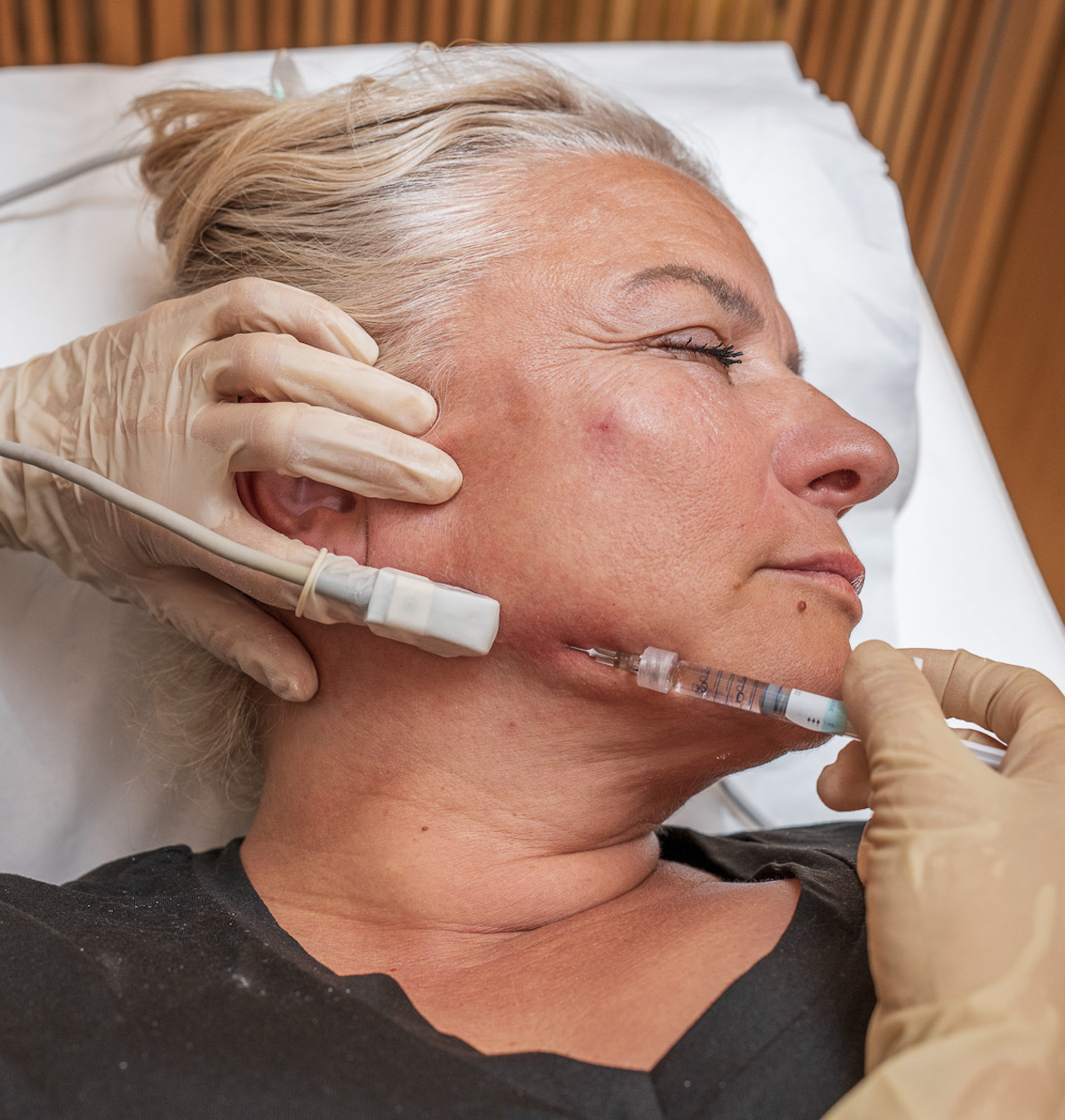

Supplement: ojag046_Supplementary_Data [file ojag046_Supplementary_Data.zip › Supplemental Figure 16A.jpg]

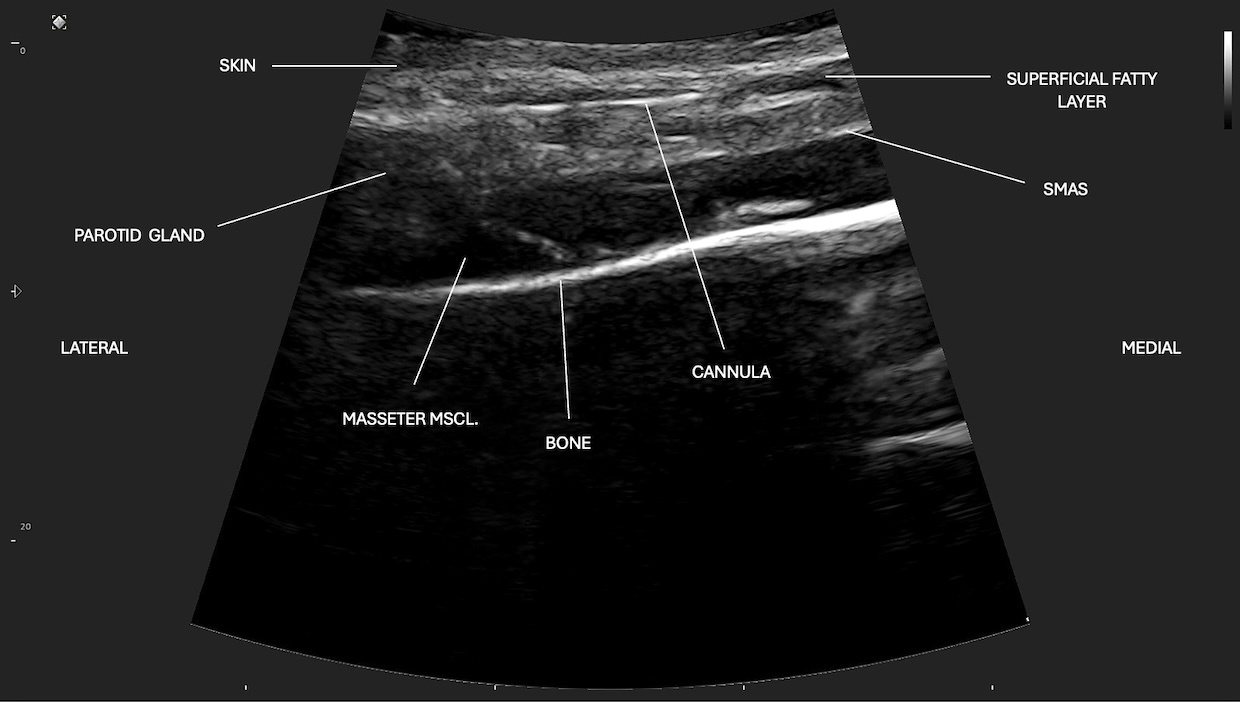

Supplement: ojag046_Supplementary_Data [file ojag046_Supplementary_Data.zip › Supplemental Figure 16B.jpg]

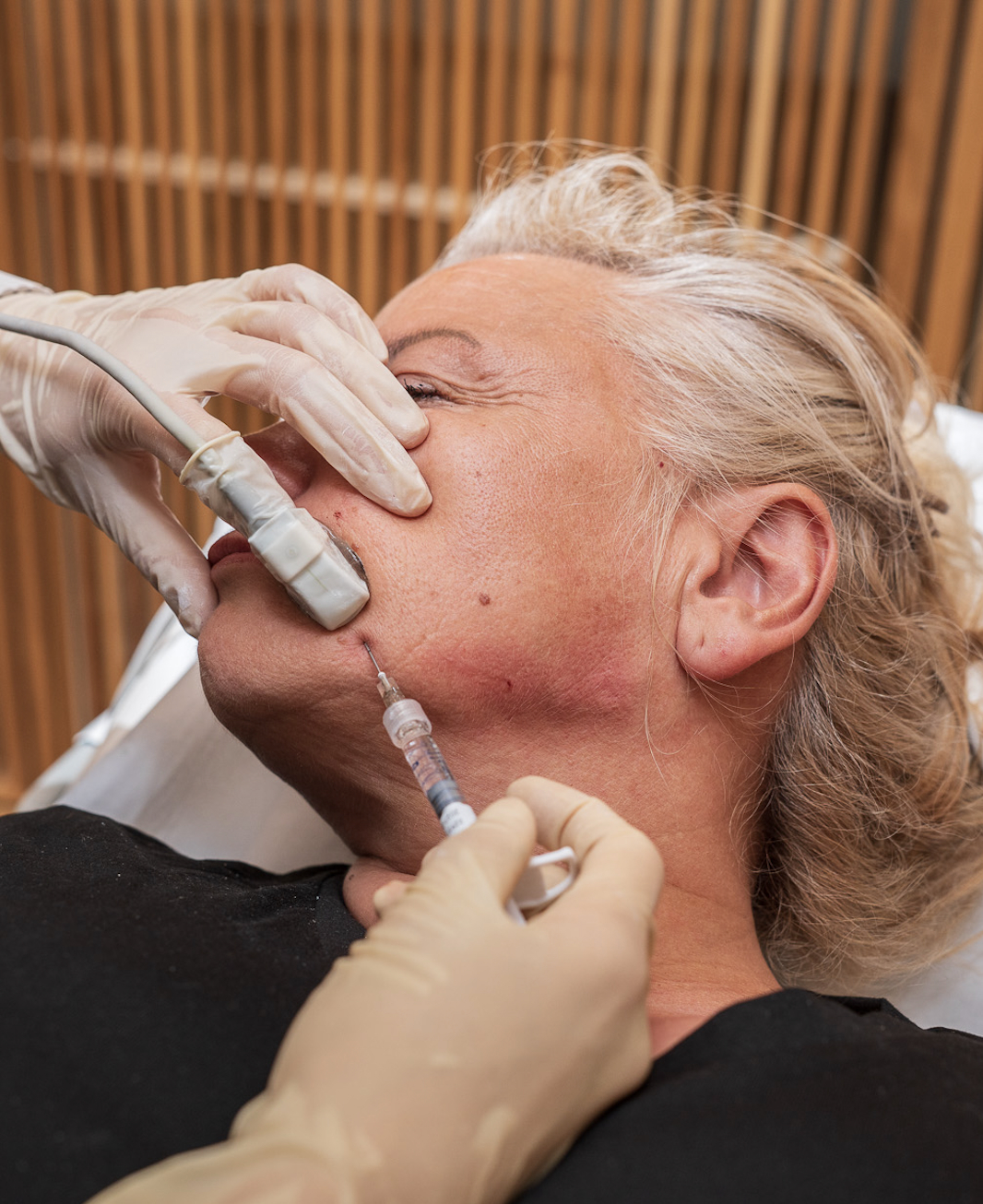

Supplement: ojag046_Supplementary_Data [file ojag046_Supplementary_Data.zip › Supplemental Figure 17A.jpg]

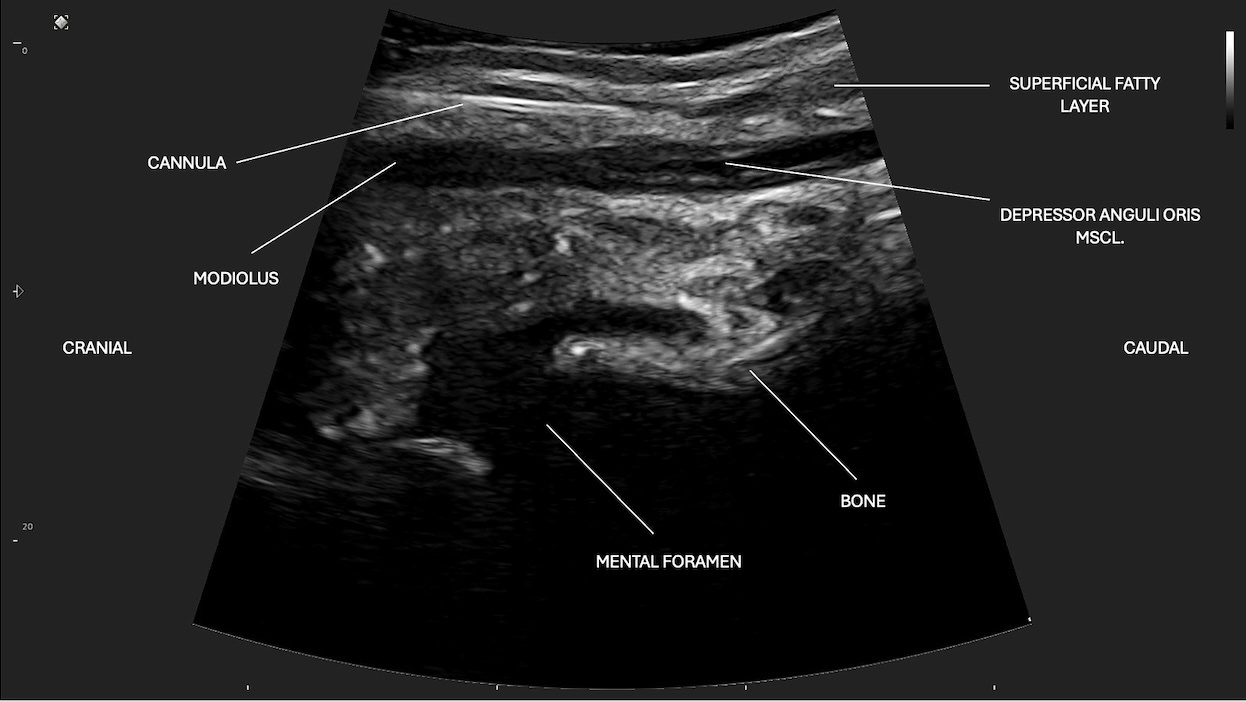

Supplement: ojag046_Supplementary_Data [file ojag046_Supplementary_Data.zip › Supplemental Figure 17B.jpg]

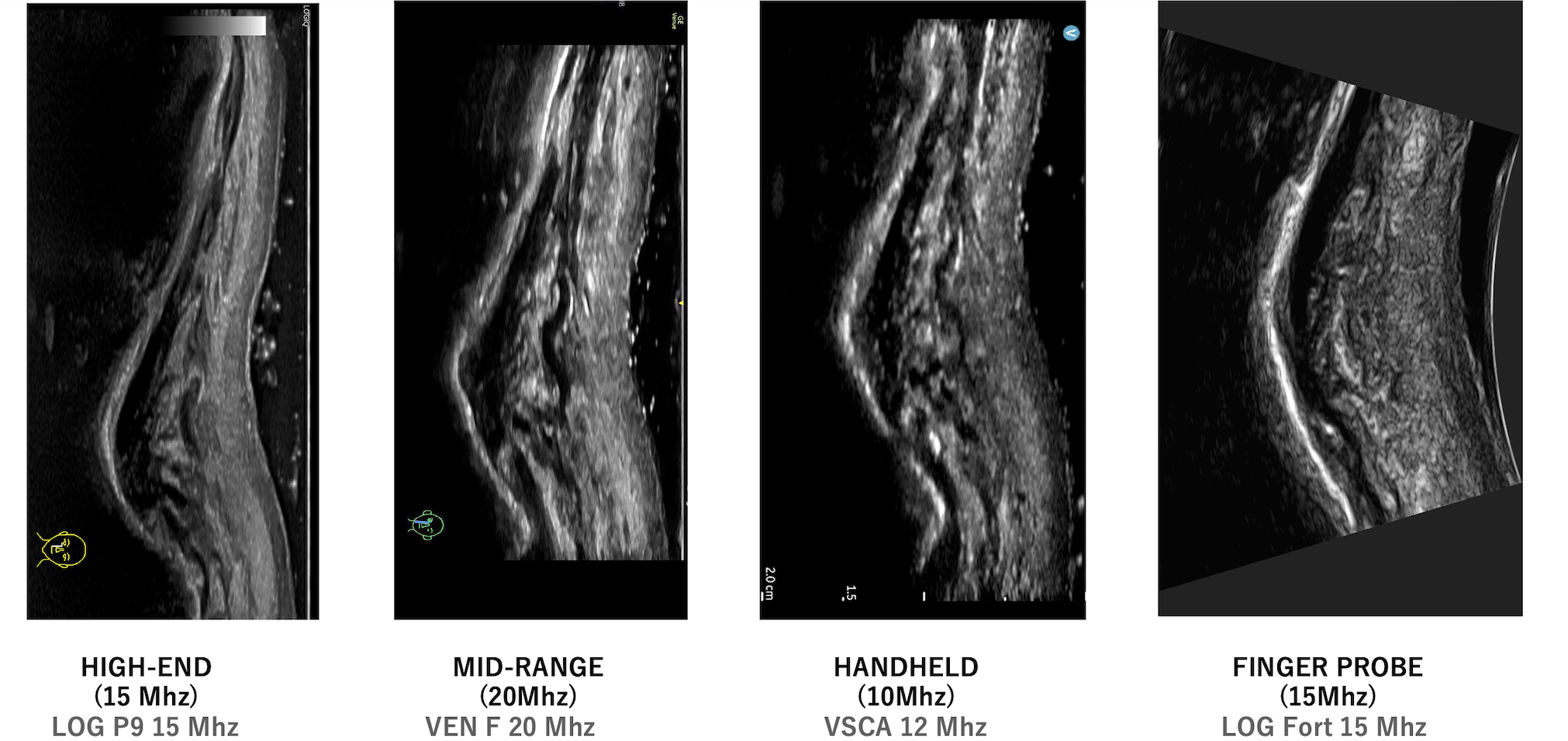

Supplement: ojag046_Supplementary_Data [file ojag046_Supplementary_Data.zip › Supplemental Figure 18.jpg]

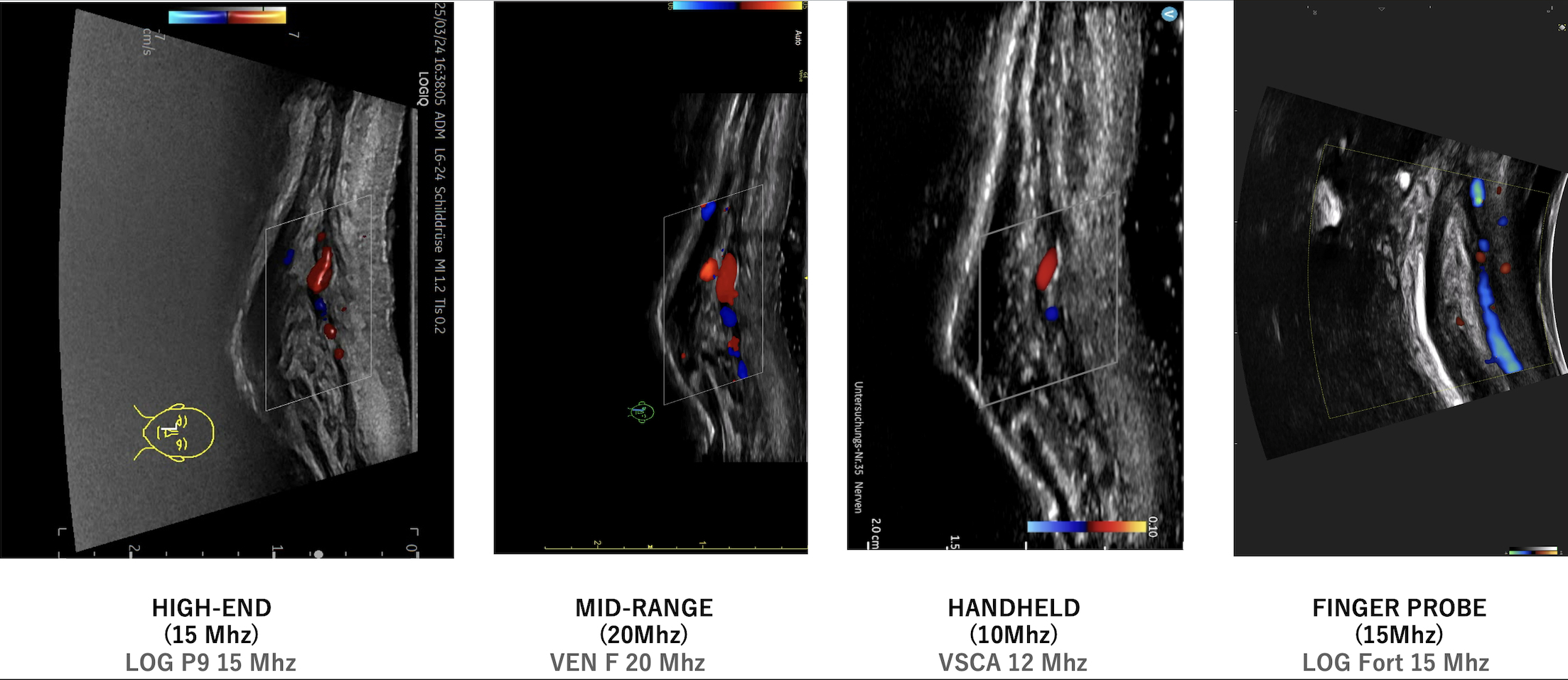

Supplement: ojag046_Supplementary_Data [file ojag046_Supplementary_Data.zip › Supplemental Figure 19.jpg]

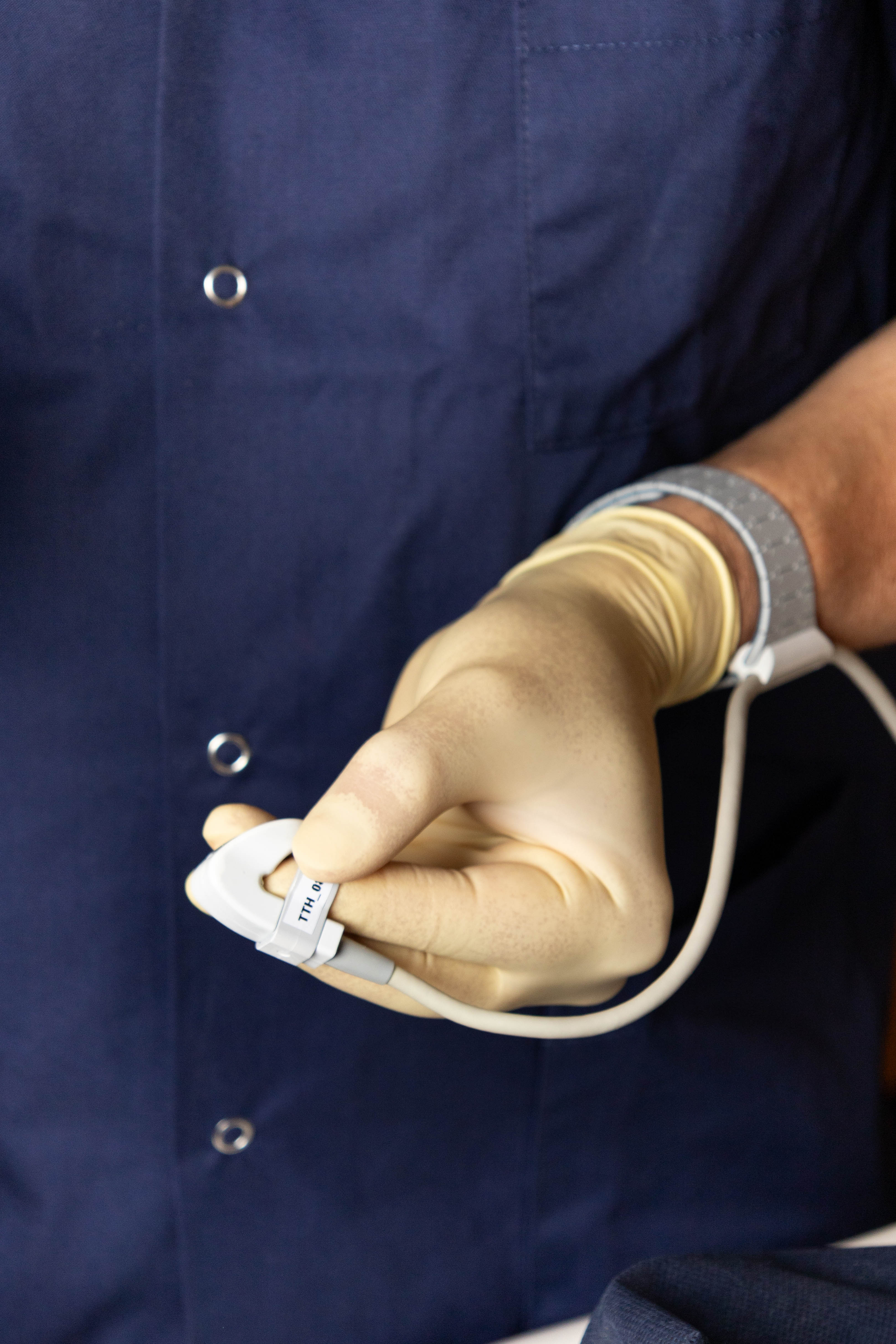

Supplement: ojag046_Supplementary_Data [file ojag046_Supplementary_Data.zip › Supplemental Figure 2.jpg]

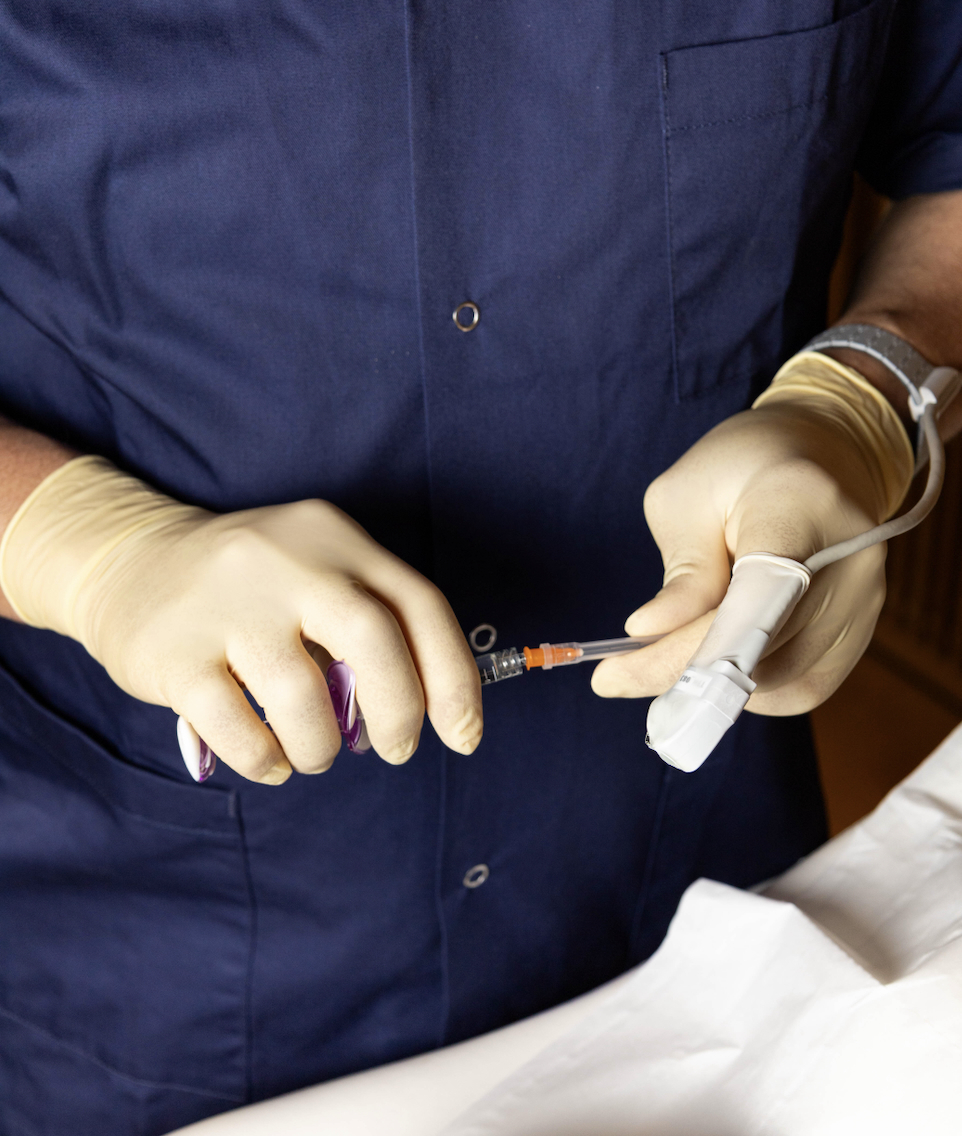

Supplement: ojag046_Supplementary_Data [file ojag046_Supplementary_Data.zip › Supplemental Figure 3.jpg]

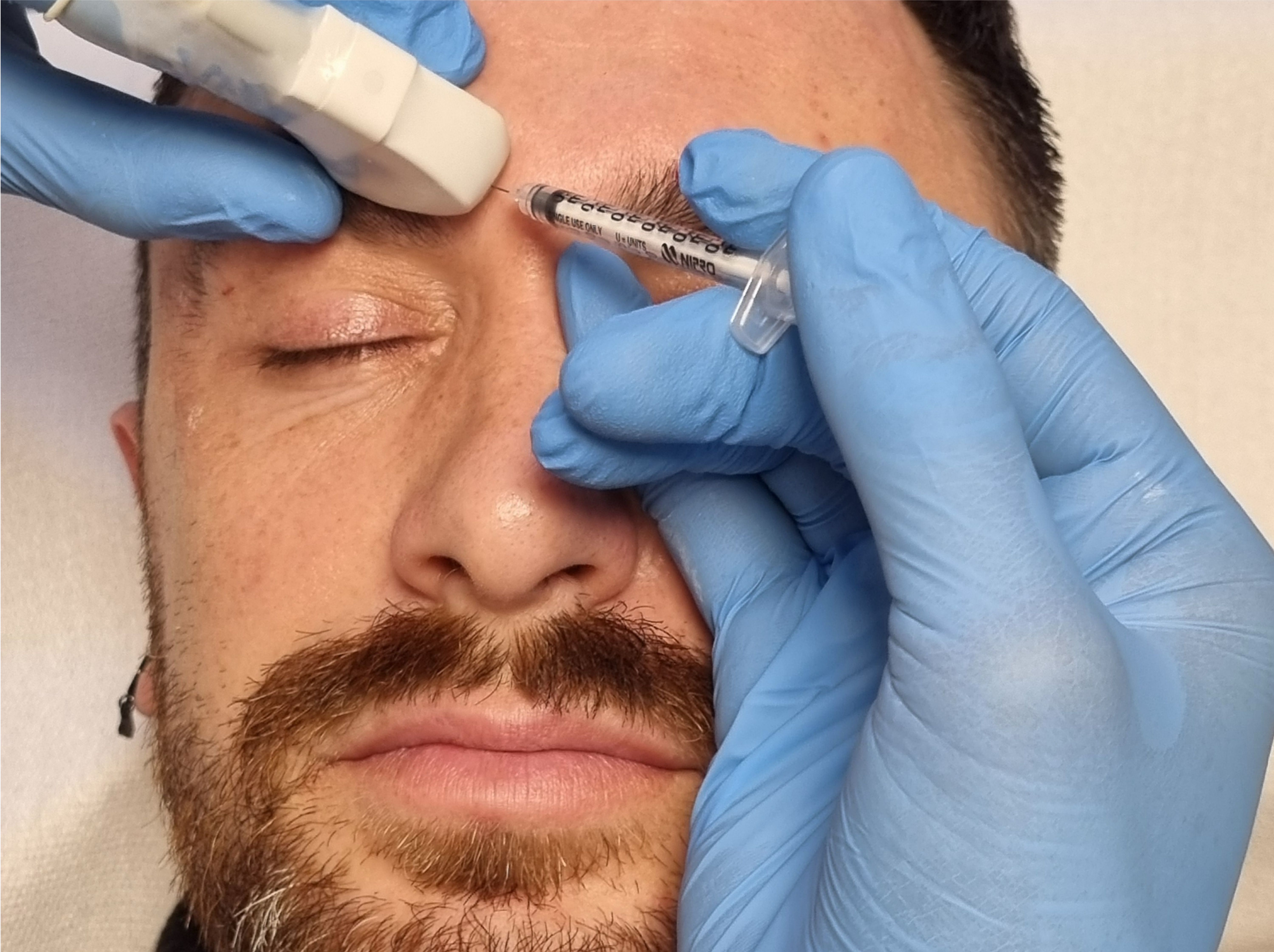

Supplement: ojag046_Supplementary_Data [file ojag046_Supplementary_Data.zip › Supplemental Figure 4A.jpg]

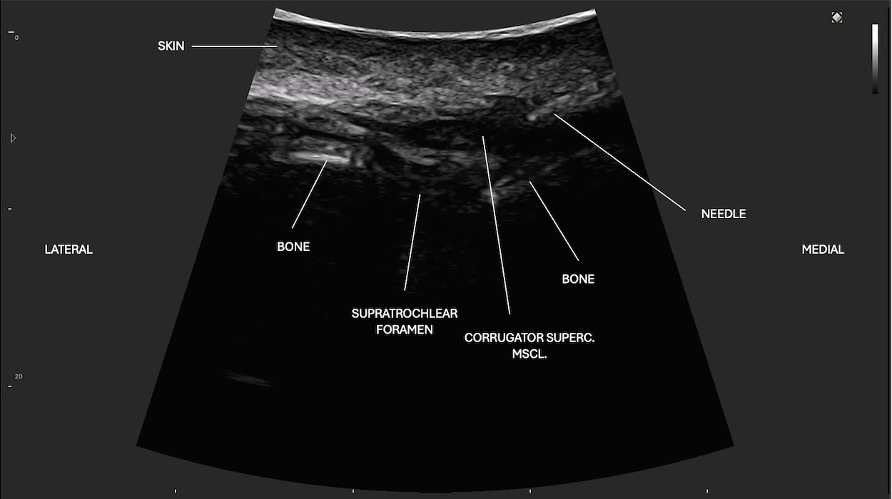

Supplement: ojag046_Supplementary_Data [file ojag046_Supplementary_Data.zip › Supplemental Figure 4B.jpg]

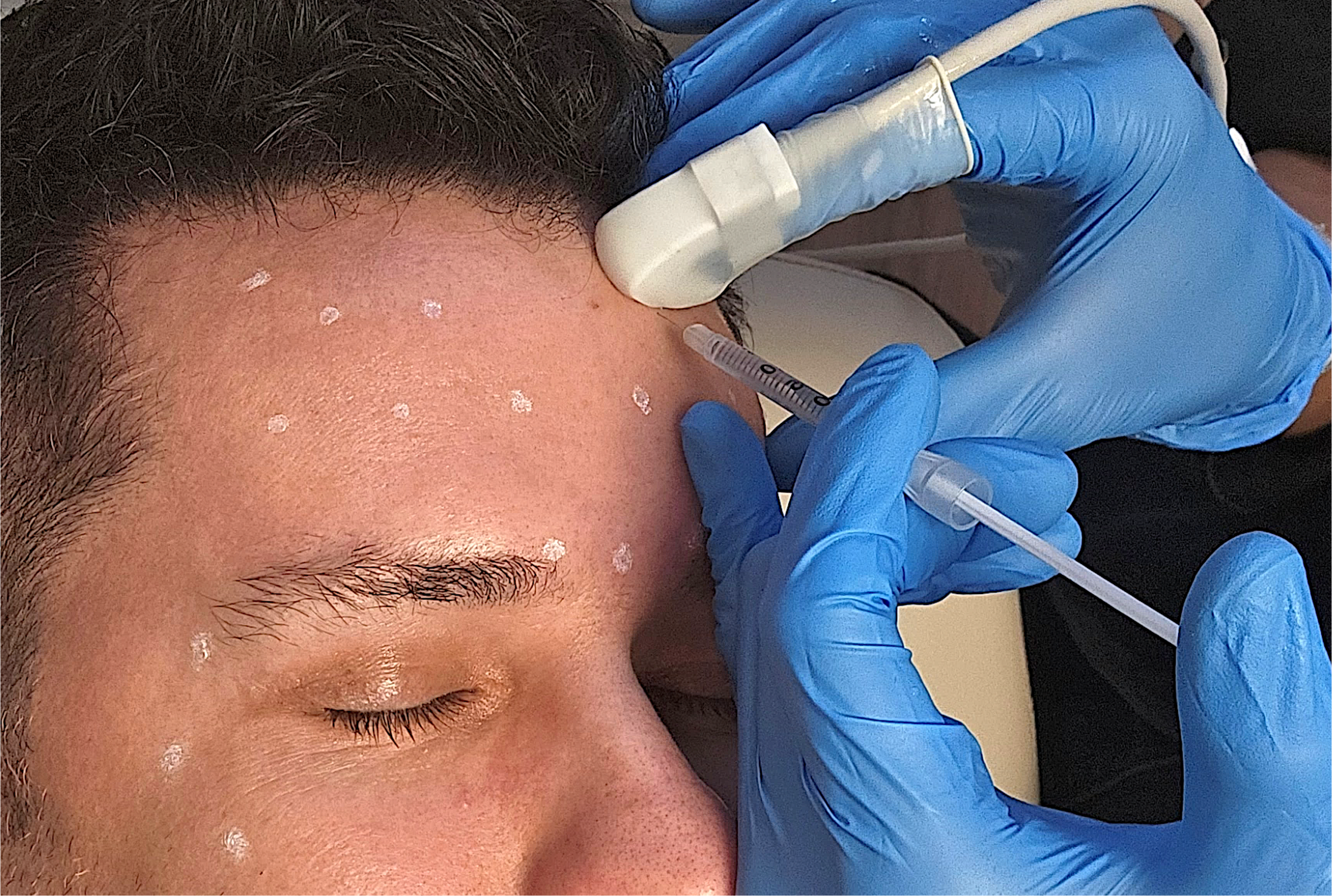

Supplement: ojag046_Supplementary_Data [file ojag046_Supplementary_Data.zip › Supplemental Figure 5A.png]

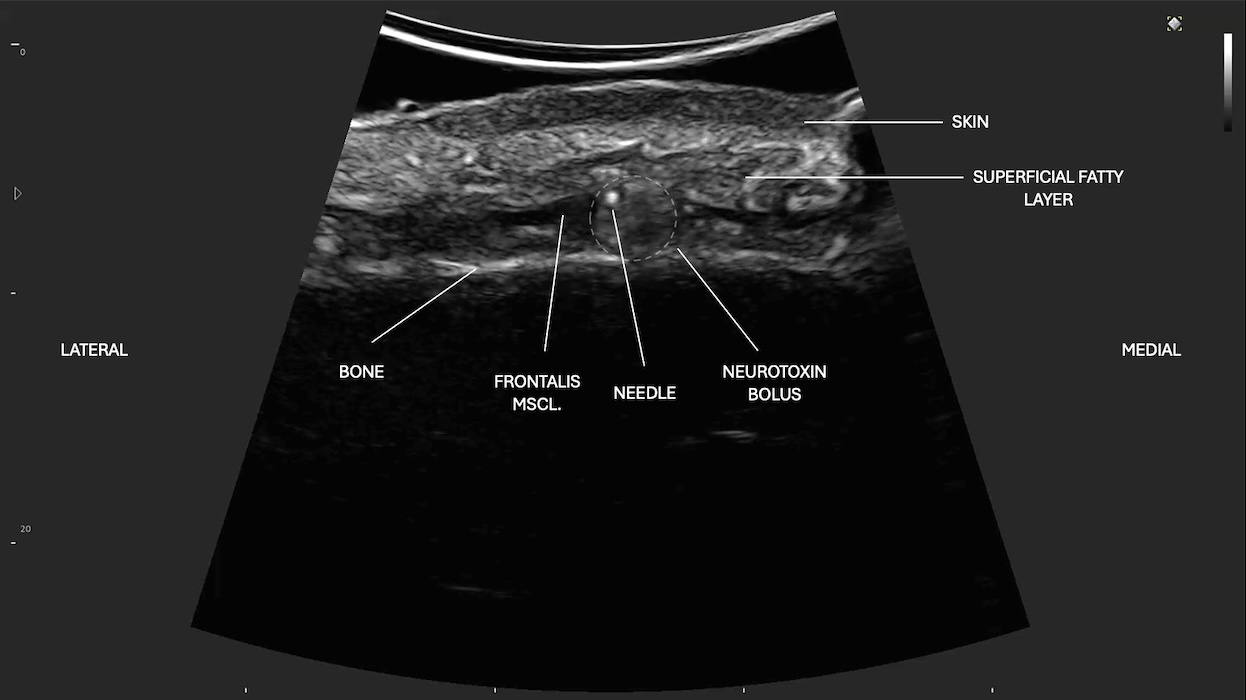

Supplement: ojag046_Supplementary_Data [file ojag046_Supplementary_Data.zip › Supplemental Figure 5B.jpg]

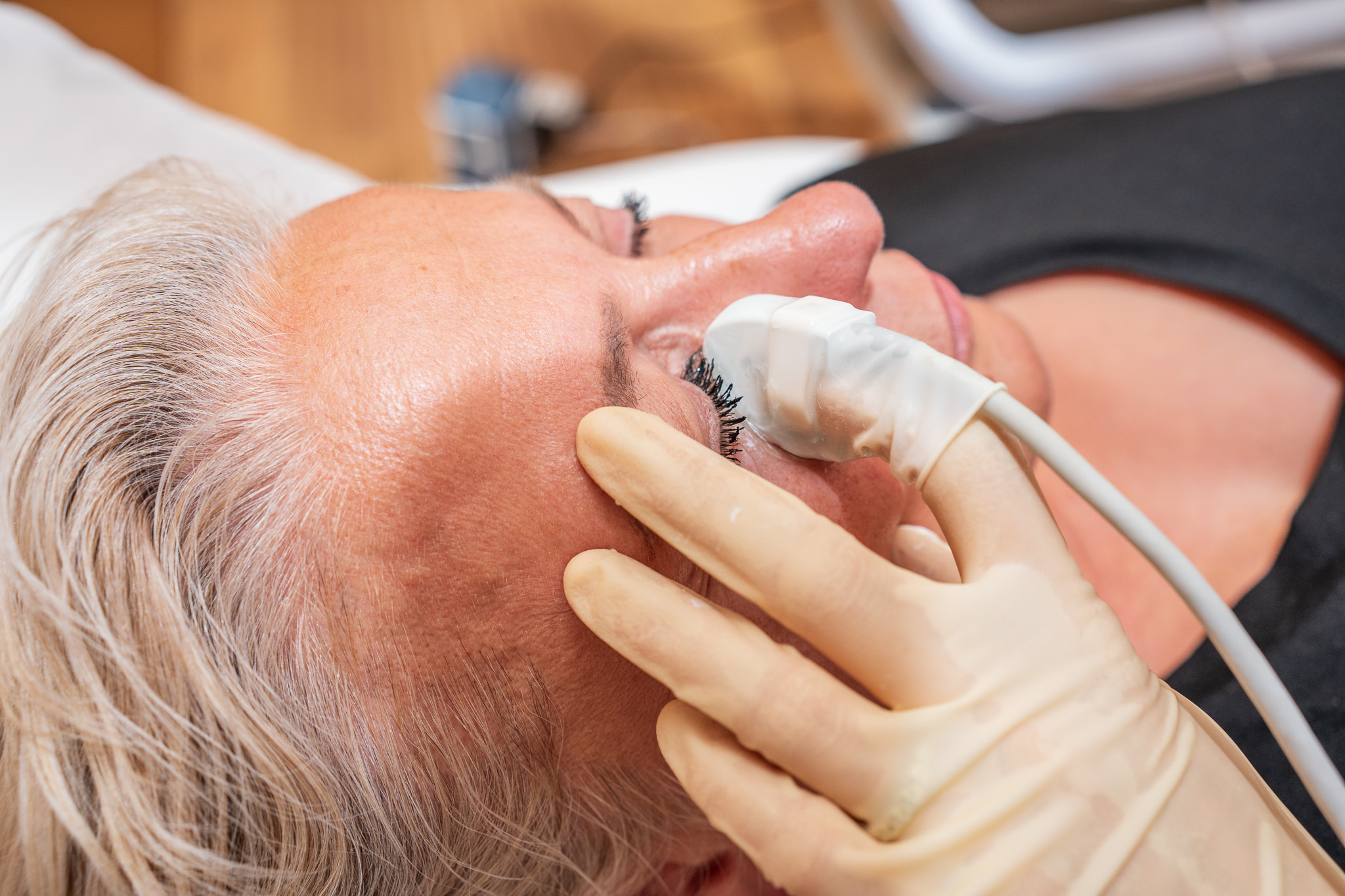

Supplement: ojag046_Supplementary_Data [file ojag046_Supplementary_Data.zip › Supplemental Figure 6A.jpg]

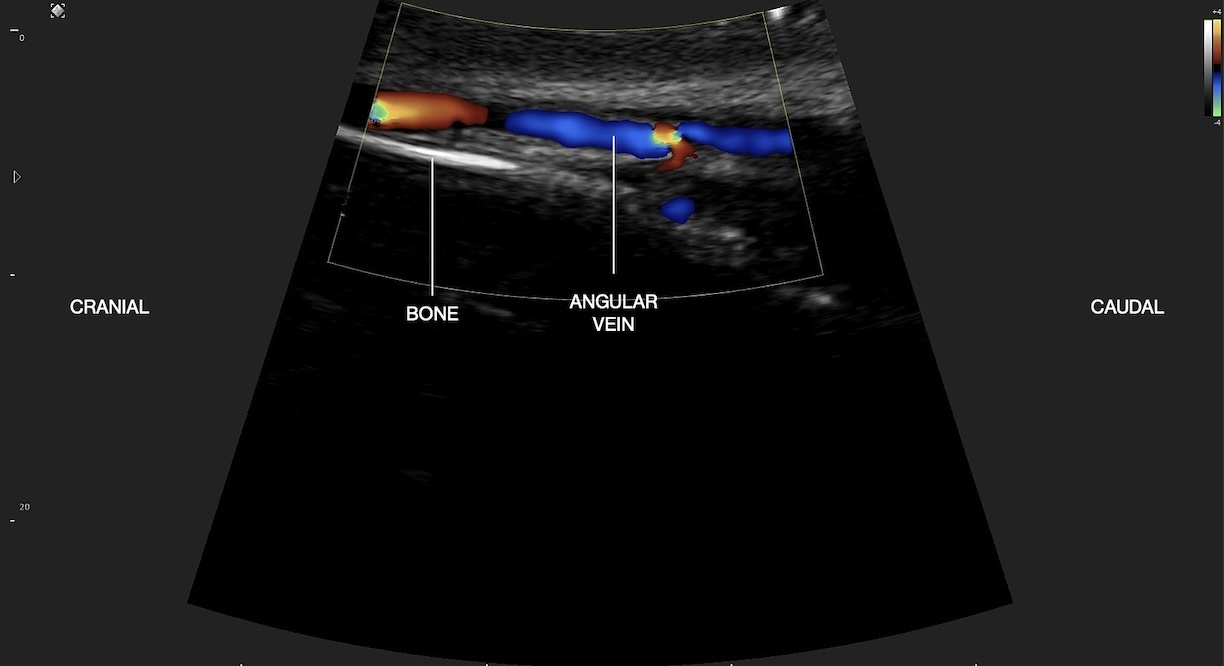

Supplement: ojag046_Supplementary_Data [file ojag046_Supplementary_Data.zip › Supplemental Figure 6B.jpg]

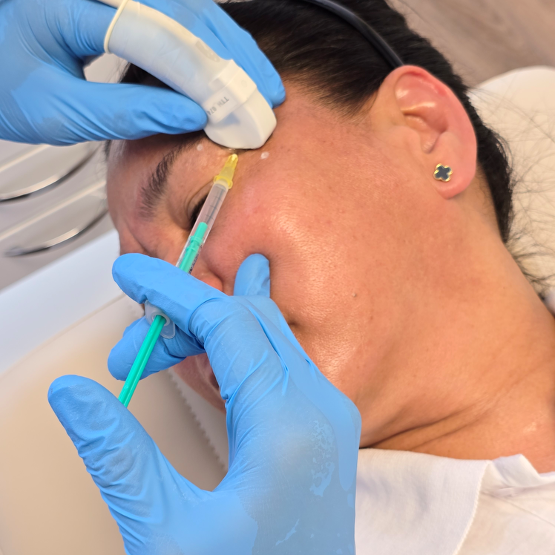

Supplement: ojag046_Supplementary_Data [file ojag046_Supplementary_Data.zip › Supplemental Figure 7A.png]

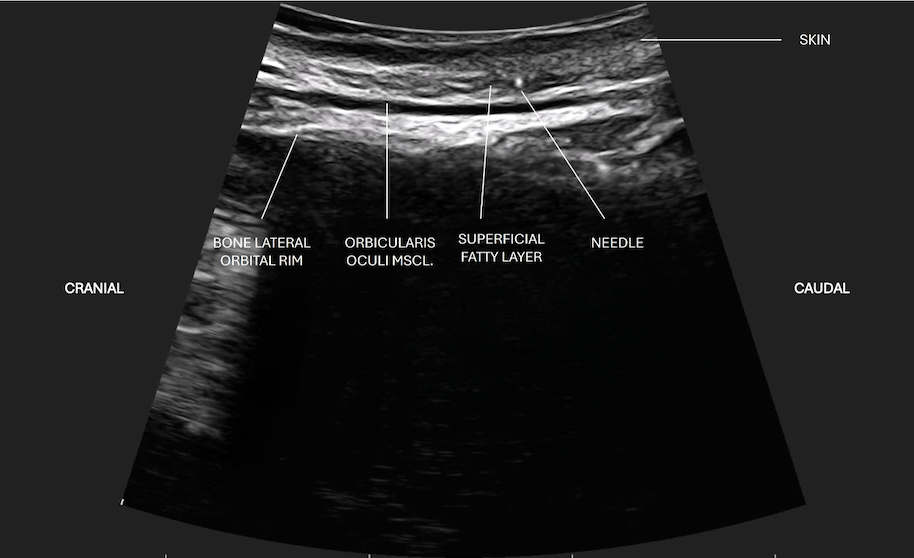

Supplement: ojag046_Supplementary_Data [file ojag046_Supplementary_Data.zip › Supplemental Figure 7B.jpg]

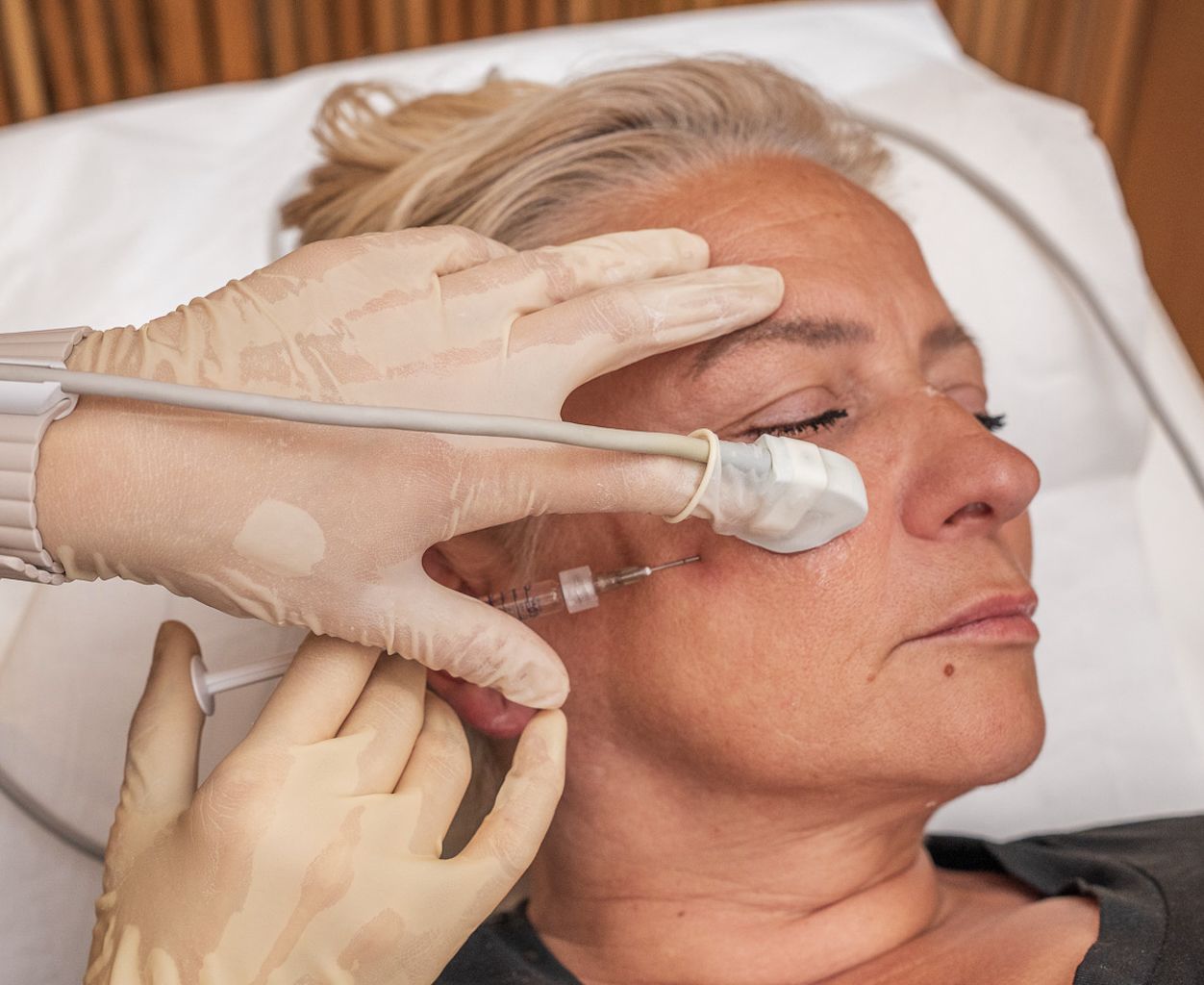

Supplement: ojag046_Supplementary_Data [file ojag046_Supplementary_Data.zip › Supplemental Figure 8A.jpg]

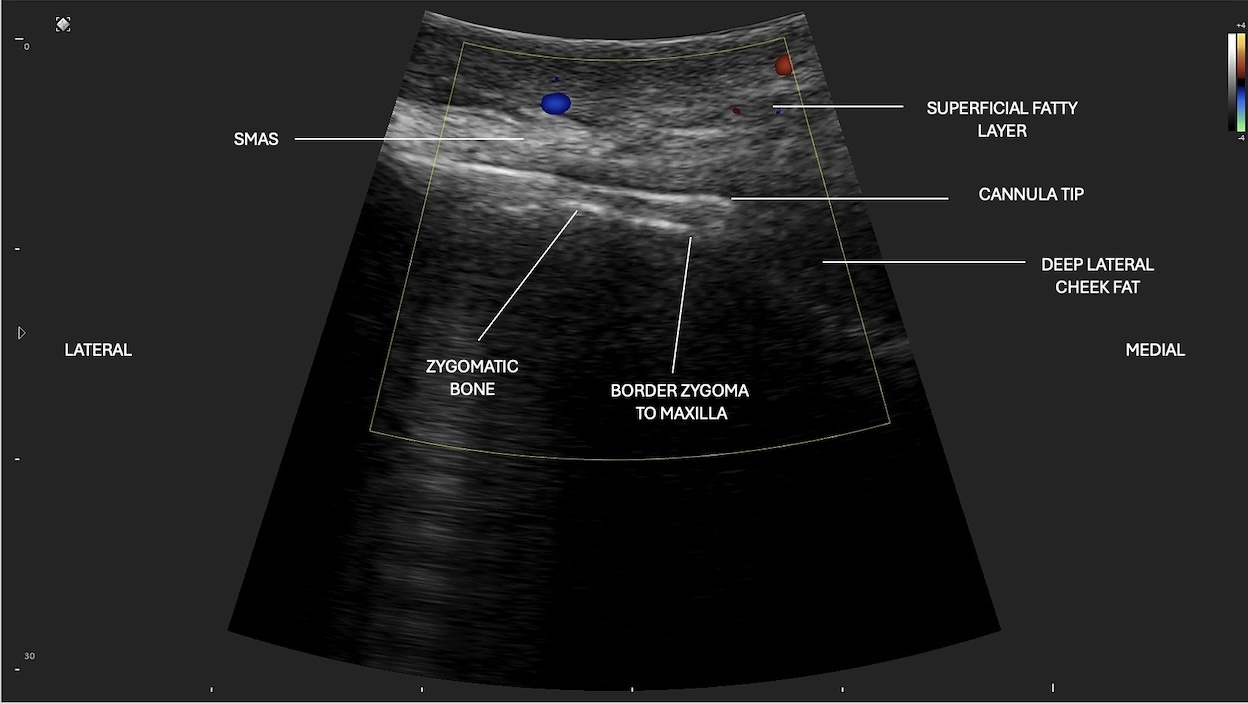

Supplement: ojag046_Supplementary_Data [file ojag046_Supplementary_Data.zip › Supplemental Figure 8B.jpg]

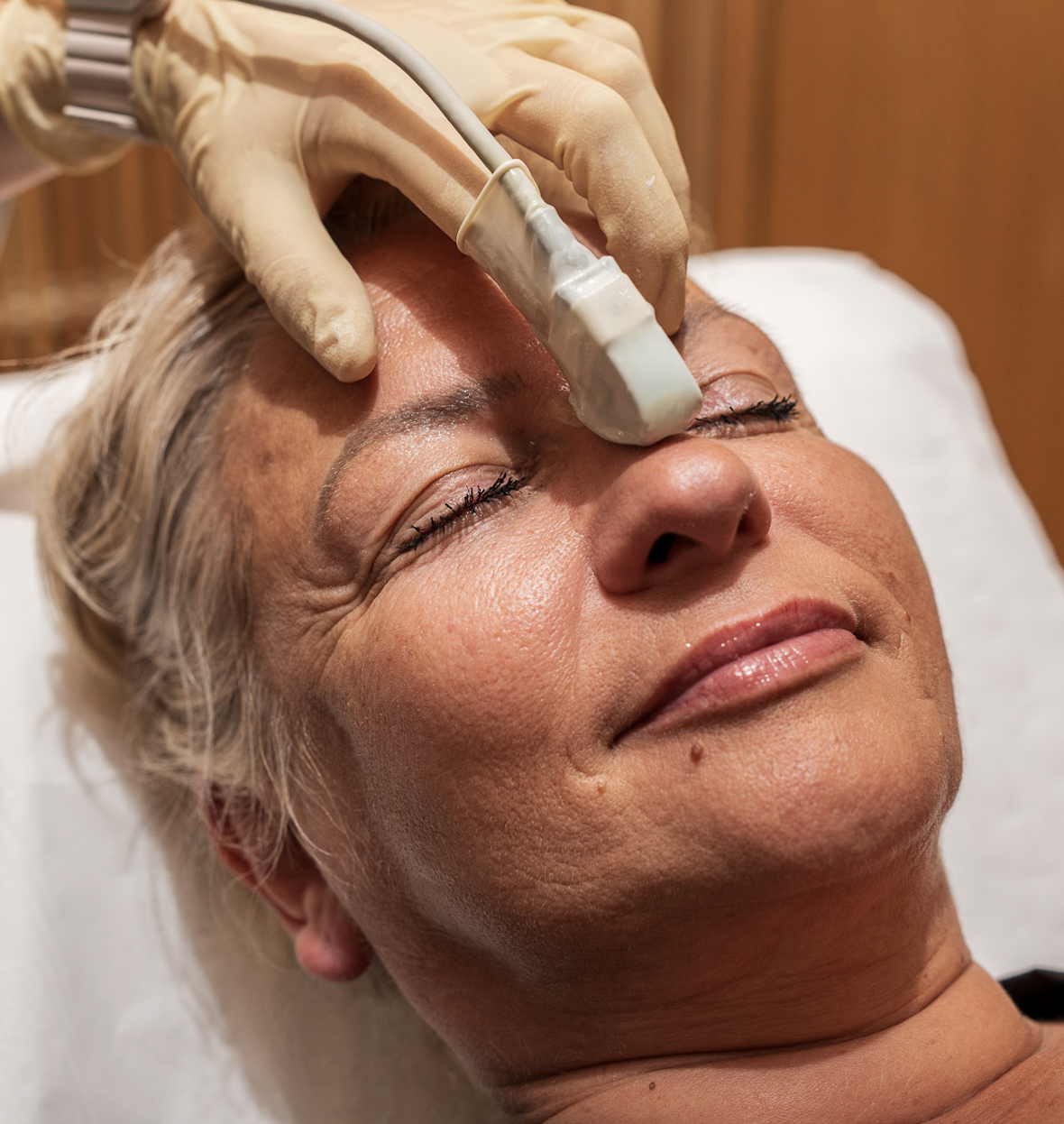

Supplement: ojag046_Supplementary_Data [file ojag046_Supplementary_Data.zip › Supplemental Figure 9A.jpg]

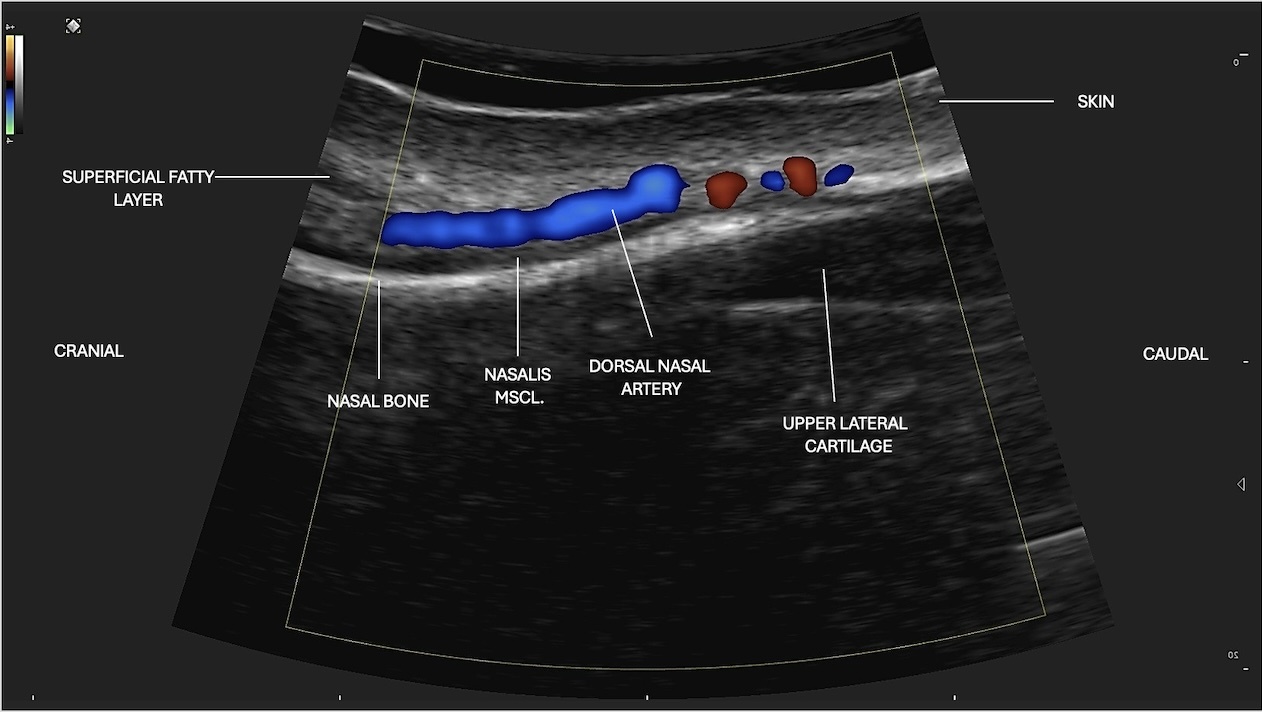

Supplement: ojag046_Supplementary_Data [file ojag046_Supplementary_Data.zip › Supplemental Figure 9B.jpg]
